# Investigating the Mechanism of Antimycobacterial and Antiproliferative Activity of (*E*)‐*N*’‐Benzylidenepyrazine‐2‐Carbohydrazides and their Derivatives

**DOI:** 10.1002/cmdc.202500085

**Published:** 2025-08-29

**Authors:** Priam‐Amedeo Houngbedji, Daria Elżbieta Nawrot, Ondřej Janďourek, Klára Konečná, Martin Novák, Pavla Paterová, Pavel Bárta, Martina Hrast Rambaher, Eva Novotná, Carlo Castellano, Matteo Mori, Fiorella Meneghetti, Monika Záhorszká, Jana Korduláková, Jan Zitko

**Affiliations:** ^1^ Faculty of Pharmacy in Hradec Králové Charles University Ak. Heyrovského 1203 500 03 Hradec Králové Czech Republic; ^2^ Biomedical Research Centre University Hospital Hradec Králové Sokolská 581 500 05 Hradec Králové Czech Republic; ^3^ Department of Clinical Microbiology University Hospital Hradec Králové Sokolská 581 500 05 Hradec Králové Czech Republic; ^4^ Faculty of Pharmacy University of Ljubljana Aškerčeva cesta 7 SI‐1000 Ljubljana Slovenia; ^5^ Department of Chemistry University of Milan Via Golgi 19 20133 Milan Italy; ^6^ Department of Pharmaceutical Sciences University of Milan Via Mangiagalli 25 20133 Milan Italy; ^7^ Department of Biochemistry Faculty of Natural Sciences Comenius University in Bratislava Mlynská dolina, Ilkovičova 6 842 15 Bratislava Slovakia

**Keywords:** antitumor agents, antimycobacterials, iron chelators, medicinal chemistry, pyrazine‐2‐carbohydrazides

## Abstract

A series of 33 (*E*)‐*N*’‐benzylidenepyrazine‐2‐carbohydrazides and their derivatives were synthesized and tested for biological activity. Benzylidene derivatives with 2‐OH substitution on the phenyl ring (**18**: *R* = 2‐OH, **21**: *R* = 2,3‐diOH, and **22**: *R* = 2,4‐diOH) exhibit various biological activities. Compounds **18** and **21** demonstrate antimycobacterial activity against *Mycobacterium tuberculosis* H37Ra, *M. tuberculosis* H37Rv, and *M. aurum,* with minimum inhibitory concentration values ranging from 15.625 to 62.5 μg mL^−1^. Compounds **18**, **21**, and **22** show mild cytotoxicity on several human cell lines (IC_50_ ranging from 70.2 to 500 μM). Crystallographic studies confirm the (*E*)‐configuration of compound **18** and a nearly planar molecular conformation. Due to their structural similarity with salicylaldehyde isonicotinoyl hydrazone (SIH), a known iron chelator, selected compounds were tested for iron‐chelating properties, revealing comparable or superior activity. Mechanistic assays targeting enoyl‐[acyl carrier protein] reductase (InhA), isocitrate lyase (ICL), and lipid/mycolic acid biosynthesis show no significant inhibition, suggesting a nonspecific mechanism potentially linked to iron chelation. A correlation is observed between chelating activity and cytotoxicity, while antimycobacterial activity appears to involve additional mechanisms. Pharmacokinetic studies with compound **18** reveal no specific plasma metabolites, and no significant metabolites are detected after incubation with human liver microsomes.

## Graphical abstract

(*E*)‐*N*’‐benzylidenepyrazine‐2‐carbohydrazides with 2‐OH, 2,3‐diOH or 2,4‐diOH substitution exhibit good antimycobacterial or antiproliferative activity, and iron chelation similar to salicylaldehyde isonicotinoyl hydrazone (SIH).

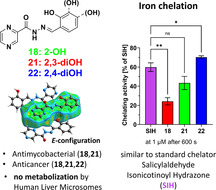

## 1 Introduction

Tuberculosis (TB), a disease caused by *Mycobacterium tuberculosis* (Mtb), causes up to 10 million new cases per year, especially in low‐ncome countries.^[^ ^1^ ^,^ ^2^ ^]^ Although the disease is curable, TB is still responsible for the death of 1.2–1.5 million people annually and is a significant contributor to antimicrobial resistance.^[^ ^1^ ^,^ ^2^ ^]^ Pyrazinamide (PZA) has been a key component of antitubercular treatment since its introduction in 1950.^[^ ^3^ ^]^ The mechanism of action of PZA is still under extensive research, with several discoveries defining PZA as a prodrug, metabolized by the mycobacterial enzyme pyrazinamidase (PncA) into pyrazinoic acid (POA).^[^ ^4^ ^]^ POA is linked with inhibitory activity on the aspartate decarboxylase (PanD), an enzyme catalyzing the transformation of aspartate into *β*–alanine, itself a precursor of pantothenate (vitamin B5) and coenzyme A. Interaction of POA with PanD also induces the degradation of the enzyme.^[^ ^5^ ^]^

One of the ways to fight mycobacterial resistance is the structural modification of antitubercular compounds already used in clinical practice.^[^ ^6^ ^]^ These structural derivatives may possess different mechanisms of action, potentially bypassing bacterial resistance.^[^ ^6^ ^]^ (*E*)‐*N’*‐benzylidenepyrazine‐2‐carbohydrazides can be considered derivatives with a modified and extended linker, derived from previously reported antimycobacterial *N*‐phenylpyrazin‐2‐carboxamides^[^ ^7^ ^]^ or isosteric *N*‐phenylpyridine‐2‐carboxamides.^[^ ^8^ ^]^ (*E*)‐*N’*‐benzylidenepyrazine‐2‐carbohydrazides reported by Vergara et al.^[^ ^9^ ^]^ exerted only weak inhibitory activity against Mtb H37Rv (ATCC 27294), with minimum inhibitory concentration (MIC) values ranging from 50 to 100 µg mL^−1^. Rodrigues et al.^[^ ^10^ ^]^ reported the testing of 51 derivatives on four cancer cell lines (HCT‐116, OVCAR 8, HL‐60, and SF‐295), showing cytotoxicity values (IC_50_ from 1.1 to 5.6 µg mL^−1^) with 2‐OH substitution favoring the antiproliferative activity. Although other works studied the biological activity of (*E*)‐*N’*‐benzylidenepyrazine‐2‐carbohydrazide series,^[^ ^11^, ^12^ ^–^ ^13^ ^]^ the results varied, and no mechanism of action has been confirmed.

**Figure 1:**
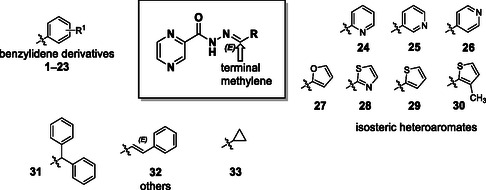
The structure of compounds investigated in this study. Substituents R^1^ for the benzylidene compounds: **1**: 2‐Cl; **2**: 3‐Cl; **3**: 4‐Cl; 4: 2‐Br; **5**: 3‐Br; **6**: 4‐Br; **7**: 2‐F; **8**: 3‐F; **9**: 4‐F; **10**: 2,3,6‐triCl; **11**: 2‐NO_2_; **12**: 3‐NO_2_; **13**: 4‐NO_2_; **14**: 2‐OMe; **15**: 4‐OH, 3‐OMe; **16**: 3‐OMe; **17**: 4‐OMe; **18**: 2‐OH; **19**: 3‐OH; **20**: 4‐OH; **21**: 2,3‐diOH; **22**: 2,4‐diOH; and **23**: 4‐OH, 3‐NO_2_.

Our current work aims at broadening the palette of available (*E*)‐*N’*‐benzylidenepyrazine‐2‐carbohydrazides (**Figure** **1**, **1**–**23**), providing more complete data about their antimicrobial and antiproliferative activity, and studying the potential mechanism of action of selected panel compounds. To extend the chemical space around the title derivatives, we also prepared similar derivatives with various substituents on the terminal methylene group—mainly heterocyclic isosteres of the benzylidene compounds (**24**–**30**). To identify a possible mechanism of action, an in vitro iron chelation assay was conducted with selected title compounds motivated by their structural similarity with salicylaldehyde isonicotinoyl hydrazone (SIH), a well‐documented iron chelator.^[^ ^14^ ^]^ To explore alternative explanations for the observed biological activity, inhibition assays were conducted on several enzymatic targets linked with antimycobacterial activity, such as enoyl‐ACP‐reductase (InhA) and isocitrate lyase (ICL). Parallelly, the effect of selected compounds on mycolic acid and lipid synthesis in Mtb H37Rv was studied. The results of these studies bring more information about the relation between the structure and the biological activity of (*E*)‐*N’*‐benzylidenepyrazine‐2‐carbohydrazides, and move the current state of art from biological activity testing toward the identification of a mechanism of action.

## 2 Results and Discussion

### 2.1 Chemistry

**Scheme 1:**
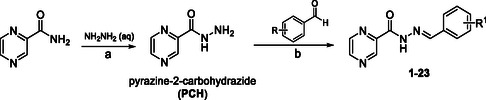
General synthetic approach for benzylidene derivatives **1**–**23**. Conditions: a–1. THF, reflux, 12 h, 2. EtOH, reflux, 12 h; b–MeOH, 70 °C, 3 h. Note: Derivatives **24**–**33** were prepared analogously using the corresponding aldehydes.

The synthesis of the reported series started with the preparation of pyrazine‐2‐carbohydrazide (PCH) by stirring PZA with an excess of hydrazine in tetrahydrofuran at 70 °C under reflux (**Scheme** **1**, a).^[^ ^15^ ^]^ This led to nucleophilic substitution at the carbonyl of the amidic functional group. After the reaction, the solvents and the excess hydrazine were removed on a rotary evaporator. The crude product was stirred and refluxed in EtOH overnight to remove excess hydrazine. The reaction mixture was left to cool down to room temperature to acquire the precipitate, which was filtered off, dried and used for the subsequent reactions without further purification. PCH was combined with respective aldehydes in MeOH and mixed at elevated temperature (60–70 °C) for 3 h (Scheme 1, b). The heating was then turned off and the solution was left to stir until a precipitate appeared. The precipitate was filtered and washed with cold MeOH to yield final compounds **1**–**33**.

All compounds were isolated as solids, with yields ranging from 80 to 96% (the final step). Compounds were characterized by ^1^H NMR and ^13^C NMR spectra, elemental analysis, and melting points. Pivotal compounds (**18**, **21**, and **22**), defined as compounds with significant detected antimycobacterial activity—see section 2.3.1., were further characterized by IR spectroscopy (ATR‐Ge). The identity and purity of fluorinated compounds (**7**–**9**) and pivotal compounds of this study (**18**, **21**, and **22**) were additionally checked by high‐performance liquid chromatography–high‐resolution mass spectrometry (HPLC–HRMS). All analytical data were consistent with the proposed structures. Characteristic signals in ^1^H NMR (hydrazide proton) appeared between 12.05 and 12.73 ppm when measured in DMSO. The full characterization of compounds is located in Supporting Information.

The obtained IR spectra showed the following characteristic signals: 3330–3120 (phenolic ‐OH stretch), 3050–3290 (amidic ‐NH stretch), 1670–1660 (C=O), 1635–1605 (C=N), 1530–1500 (N—N), 1465–1355 (aromatic C—C stretch).

### 2.2 Crystal Structure of Compound 18

**Figure 2:**
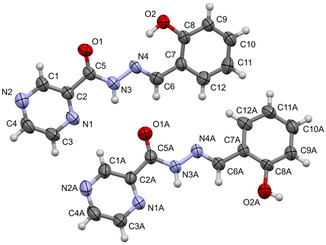
Thermal ellipsoid diagram of **18**, with the arbitrary atom‐numbering scheme. Displacement ellipsoids are drawn at the 50% probability level. In the following text, the upper molecule is designated as I, and the lower molecule as A.

The pivotal 2‐OH benzylidene derivative **18** was analyzed by single‐crystal X‐ray diffraction to study the configuration of the double bond and define the 3D arrangement of this class of compounds. The unit‐cell determination revealed a monoclinic P21/c space group, with two molecules in the asymmetric unit (ASU), differing in the orientation of their phenol moiety. The thermal ellipsoid diagram, indicating the arbitrary atom‐numbering scheme used in the discussion, is reported in **Figure** **2**.

The overall structure is almost planar, with the phenol moiety and the pyrazine ring inclined at 2.5° and 5.5° with respect to each other in molecules I and A, respectively. The analysis confirmed the absolute configuration (*E*) on the C=N double bond in the linker. The phenyl ring is present in two distinct conformations, with the 2‐OH group facing the same or the opposite direction with respect to the ‐NH‐ hydrogen of the carbohydrazide linker. These findings correlate with crystallographic studies previously reported in the literature.^[^ ^16^ ^]^ Moreover, all bond lengths, valence angles, and torsional angles are within the expected limits for similar compounds, as shown by CSD Mogul.^[^ ^17^ ^]^ The detailed description of the structure, including the crystal packing (Figure S2, Supporting Information), observed hydrogen bonds (Table S4, Supporting Information), and visualization of the Hirshfeld surface (Figure S3), is located in the Supporting Information.

### 2.3 Antimicrobial and Cytostatic Activity

#### 2.3.1 In Vitro Antimycobacterial Activity

Final compounds were tested against a selection of mycobacterial strains (Mtb H37Ra, Mtb H37Rv, *M. smegmatis*, and *M. aurum*) by the Microplate Alamar Blue Assay (MABA). Results expressed as minimum inhibitory concentrations (MIC) are reported in **Table** **1**.

**Table 1:** The results of antimycobacterial screening of compounds **18**, **21**, and **22** against selected mycobacterial strains expressed as minimum inhibitory concentration MIC [µg mL^−1^].

CODE	*Mycobacterium tuberculosis* H37Ra	*Mycobacterium tuberculosis* H37Rv	*Mycolicibacterium smegmatis*	*Mycolicibacterium aurum*
**18**	62.5	25	>500	31.25
**21**	15.625	25	>500	15.625
**22**	>500	n/a	>500	>500
INH	0.25	1.56	31.25	1.98
RIF	0.003	n/a	25	0.39
CIP	0.25	n/a	0.125	0.016

INH—isoniazid, RIF—rifampicin, CIP—ciprofloxacin. n/a—not available.

Only compounds with a ‐OH group in *ortho* position (**18**, **21**, **22)** on the benzylidene ring showed antimycobacterial activity. Despite containing a ‐OH substituent in *ortho* position, compound **22** showed no antimycobacterial activity (Table 1). Compounds not included in Table 1 were generally inactive with MIC > 250–500 µg mL^−1^. The detailed methodology and full results are available in Supporting Information.

#### 2.3.2 In Vitro Antibacterial and Antifungal Activity

All final compounds were screened for in vitro antibacterial and antifungal activities. None of the tested molecules showed antibacterial activity when tested against *Staphylococcus aureus*, methicillin‐resistant *Staphylococcus aureus, Staphylococcus epidermidis, Enterococcus faecalis, Escherichia coli, Klebsiella pneumoniae, Acinetobacter baumannii,* and *Pseudomonas aeruginosa* up to the highest tested concentration (500 μM). However, compounds **18** and **21** showed mild antifungal activities (see **Table** **2**). Compounds not included in Table 2 were generally inactive with MIC > 250–500 μM. The detailed methodology and full results are available in Supporting Information.

**Table 2:** The results of antifungal screening of compounds **18**, **21**, and **22**.

	Strain/MIC [µM]							
CODE	CA	CK	CP	CT	AF	AFla	LC	TI
**18**	250	125	>500	500	>500	>500	250	62.5
**21**	250	62.5	250	125	>250	>250	250	62.5
**22**	>125	>125	>125	>125	>125	>125	>125	>125

Antifungal activity is expressed as MIC (µM) determined after 24 h (72 h for TI) incubation period. Fungal strains: *Candida albicans* (CA)*, Candida krusei* (CK)*, Candida parapsilosis* (CP)*, Candida tropicalis* (CT)*, Aspergillus fumigatus* (AF)*, Aspergillus flavus* (AFla)*, Lichtheimia corymbifera* (LC)*,* and *Trichophyton interdigitale* (TI)*.* Standards and full methodology are described in Supporting Information.

#### 2.3.3 In Vitro Cytotoxicity

The cytotoxicity of the most promising compounds (**18**, **21**, and **22**) was evaluated on five cancer cell lines, namely HepG2, A498, PC‐3, SK‐OV‐3, and U‐87 MG (**Table** **3**). There was a complication with the precipitation of the compound **22** during some of the testing. The stated value over 100 µM indicates the minimal concentration at which no precipitation occurred.

**Table 3:** In vitro cytotoxicity of compounds **18**, **21**, and **22** expressed as IC_50_ (μM).

CODE	Cell linea)				
	HepG2	A498	PC‐3	SK‐OV‐3	U‐87 MG
**18**	241.6	440.2	396.2	383.7	≥500
**21**	143.3	186.6	160.5	248.9	223.7
**22**	70.2	≥100	≥100	≥100	≥100

a) Cell lines: All cell lines are of human origin. Hepatocellular carcinoma cells HepG2, epithelial kidney carcinoma cells A498, prostate adenocarcinoma cells PC‐3, ovarian adenocarcinoma cells SK‐OV‐3, and epithelial glioblastoma cells U‐87 MG.

#### 2.3.4 Discussion of Observed Antimicrobial Activity and Cytotoxicity

The results of antimycobacterial, antibacterial, and antifungal activity screening present a relation between the biological activity of (*E)*‐*N*’‐benzylidenepyrazine‐2‐carbohydrazide derivatives and the presence of a hydroxy substituent in position 2 (or 2 and 3) on the phenyl ring. The selectivity indices (SI) = IC_50 HepG2_(μM)/MIC_Mtb_ _H37Rv_ (μM) calculated for compounds **18** and **21** are **SI**_**18**_ **= 2.34** and **SI**_**21**_ **= 1.48,** respectively. The low selectivity indices of compounds **18** and **21,** along with the presence of anticancer activity on different cancer cell lines (HepG2, A498, PC3, SKOV3, and U87 MG) of **18, 21,** and **22,** show that the pivotal compounds tend to exert a nonselective biological effect.

The antimycobacterial activity of (*E*)‐*N*’‐benzylidenepyrazine‐2‐carbohydrazide derivatives was partially explored in the work by Vergara et al.^[^ ^9^ ^]^ who reported a series of 26 derivatives, and identified a weak antimycobacterial activity (MIC against Mtb H37Rv ranging from 50 to 100 µg mL^−1^ in a comparable MABA assay) in derivatives with electron‐withdrawing substituents in position 2 or 3 of the phenyl ring (3‐Cl, 2‐NO_2,_ or 3‐NO_2_). In our testing, these derivatives were inactive (compounds **2**, **11**, and **12**, respectively). Vergara et al. have prepared but not tested the 2‐OH derivative, which was the second most active antimycobacterial compound in our testing (compound **18**).

Rodrigues et al. ^[^ ^10^ ^]^ reported the testing of 51 derivatives on four cancer cell lines (HCT‐116, OVCAR 8, HL‐60, and SF‐295) showing good cytotoxicity values (IC_50_ from 1.1 to 5.6 µg mL^−1^), with a structure–activity relationship indicating that the presence of ‐OH groups on the phenyl ring (especially in position 2) is important for anticancer activity. Therefore, it is crucial to identify the mechanism of action that enhances the biological activity of the studied compounds when a hydroxy group is present in position 2 of the phenyl ring.

### 2.4 Assays to Explore the Potential Mechanism of Action

#### 2.4.1 In Vitro Determination of Fe‐Chelating Properties

Iron is an essential micronutrient for many living organisms, including mammals and bacteria.^[^ ^18^ ^,^ ^19^ ^]^ It is an essential cofactor for many enzymes (e.g., oxygenases, hydroxylases, and transferases), with a key role in oxidative phosphorylation and energy production.^[^ ^18^ ^,^ ^20^ ^]^ In higher animals, this metal takes part in oxygen transport as a structural component of hemoglobin.^[^ ^18^ ^]^ Iron depletion is linked with fatigue, anemia (mammals), and reduced cellular activity (bacteria and mycobacteria).^[^ ^18^ ^]^

Mammals, including humans, rely on dietary uptake for their iron requirements.^[^ ^18^ ^]^ The transport and storage of Fe are assured by two biomolecules, transferrin and ferritin, respectively.^[^ ^18^ ^]^ Pathogens, including mycobacteria, often rely on Fe income from their host. In this regard, mycobacteria utilize two iron‐chelating compounds (siderophores), extracellular carboxymycobactin and intracellular mycobactin.^[^ ^18^ ^–^ ^21^ ^]^ Carboxymycobactin has the ability to remove iron from the host's transferrin and ferritin, as well as from inorganic iron sources (ferric hydroxide, ferric phosphate), and is then uptaken through the mycobacterial cell wall by energy‐dependent mechanisms.^[^ ^18^ ^,^ ^19^ ^]^ Inside the cell, iron is transferred from extracellular carboxymycobactin to intracellular mycobactin for further iron storage.^[^ ^18^ ^]^ Iron is essential for bacterial (and mycobacterial) growth, and its reduced availability is linked to the diminished ability of pathogens to grow and multiply.^[^ ^18^ ^]^ Several attempts to use iron‐chelating drugs as potential antimycobacterials have been documented. Deferiprone, used in the treatment of thalassemia, seemed to facilitate the growth of *M. avium* in macrophage culture.^[^ ^18^ ^]^ On the other hand, 2‐pyridinecarboxyaldehyde‐2‐quinolylhydrazone (PCQH) was effective at inhibiting mycobacterial growth in macrophage culture tests.^[^ ^18^ ^]^ Pyrazolopyrimidinone (PZP) attenuates mycobacterial growth as a cytoplasmic iron chelator without interrupting mycobactin‐dependent iron acquisition.^[^ ^19^ ^]^

In cancer, cancerous cells usually present rapid, uncontrolled proliferation, which is linked with increased iron requirements.^[^ ^22^ ^]^ In these cells, the increased DNA synthesis and ribonucleotide reductase activity result in higher expression of transferrin receptor 1 (tfR1), ensuring more iron enters the cells.^[^ ^22^ ^,^ ^23^ ^]^ The ability of iron chelators to form iron‐centric complexes and reduce Fe availability for cancerous cells has been linked with convincing antiproliferative activity against a wide range of cancer cell lines.^[^ ^22^ ^,^ ^23^ ^]^

**Figure 3:**
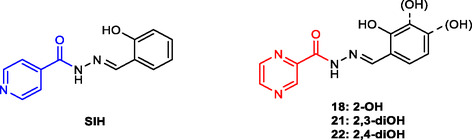
Structure comparison between salicyl isonicotinoyl hydrazone SIH and pivotal compounds **18**, **21,** and **22.** Highlighted are the isonicotinoyl **(blue)** and pyrazinoyl **(red)** moieties of otherwise similar structures.

In an attempt to elucidate the mechanism of action of 2‐OH‐benzylidene derivatives (**18**, **21**, and **22**) presenting antimycobacterial and anticancer activity, we proceeded to evaluate their in vitro iron‐chelating properties through a fluorimetry Calcein Assay following procedures reported in the literature.^[^ ^14^ ^,^ ^24^ ^]^ Calcein is a fluorescent probe that easily forms complexes with iron cations (Fe^2+^ and Fe^3+^), hence losing its fluorescence.^[^ ^14^ ^]^ The addition of iron chelators to a solution of calcein‐iron complexes removes iron from the calcein complex and restores the fluorescence.^[^ ^14^ ^]^ The iron‐chelating activity was compared to salicyl isonicotinoyl hydrazone (SIH), a well‐documented iron chelator.^[^ ^14^ ^]^ SIH is a small molecule with the ability to penetrate cell membranes and firmly bind cellular labile Fe ions.^[^ ^14^ ^]^ Its iron‐chelating properties give SIH considerable potential to protect cardiomyocytes and cardiomyoblast cells from oxidative injury.^[^ ^14^ ^]^ The use of SIH as a reference in iron‐chelation studies is further justified by its structural similarity with the studied compounds, in which the SIH isonicotinoyl moiety is replaced by pyrazinoyl (**Figure** **3**).

**Figure 4:**
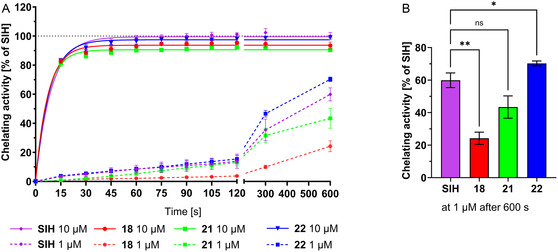
In vitro Fe‐chelating activity of selected compounds at 10 and 1 µM. Chelating activities of the tested compounds were examined by monitoring the increase of fluorescence (caused by displacement of Fe from its complex with calcein in a buffered solution (pH = 7.2). Measured activities are expressed in relation to SIH at 10 µM at time *t* = 10 min (100%). A) Chelating activity as a function of time. Full lines represent nonlinear regression (one‐phase association) for 10 µM. Dashed lines represent data for concentration of 1 µM. B) Chelating activity of tested compounds at 1 µM and time *t* = 10 min. Data are presented as mean ± SD; *n* = 4; statistical significance (one‐way ANOVA), n.s.—not significant, * *p *≤ 0.05, ** *p *≤ 0.01, and *** *p *≤ 0.001.

At the concentration of 10 µM, both SIH and the tested compounds achieved almost complete and fast restoration of fluorescence, and the differences in the kinetics of this process were insignificant. At 1 µM, the restoration of fluorescence was much slower, with a significant increase happening not sooner than after 5 min of incubation (**Figure** **4**A). At the final time point (Figure 4B), compound **18** was less effective (*p *≤ 0.01) and compound **22** was more effective (*p *≤ 0.05) than SIH. This indicates that the addition of the second hydroxyl on the benzene core might enhance the Fe chelating properties. This effect is probably not caused by the creation of another chelating site between two adjacent hydroxyls because 2,3‐diOH derivative **21** is less effective than 2,4‐diOH derivative **22**. The enhanced activity might be connected to altered electronic properties of the molecule and/or increased water solubility of such derivatives.

Although further tests are required to assess intracellular chelating properties, current data suggest that the prepared compounds have significant Fe‐chelating activity at least comparable to standard SIH. Therefore, the Fe‐chelation should always be considered for the explanation of biological activities of such compounds. In light of this finding, we reexamined the in vitro antimycobacterial activity of the compounds in conditions with increased and depleted iron in the cultivation medium (see **Table** **4**). The MIC values in normal medium were consistent with those in the original testing (Table 1 in section 2.3.1.). The only activity shift in altered conditions was observed for compound **18**, when MIC in Fe‐enriched medium increased (activity decreased) by one step on the dilution scale (Table 4). This effect could be explained by compound **18** forming an Fe‐complex, which itself could be of lower solubility/reduced ability to penetrate mycobacteria. Alternatively, the decreased activity could be perceived as the result of mycobacteria having a larger pool of Fe available. Either way, these explanations are only hypothetical because the difference in activity in normal versus Fe‐enriched medium is only minor, and in the binary dilution setup, the neighboring MIC values are often considered to be the same activity. Moreover, the MIC shift was observed for compound **18**, which had the lowest chelating properties. On the contrary, compound **22,** with the highest chelating activity, was devoid of any antimycobacterial activity. Combined, this leads to the conclusion that the antimycobacterial activity of the title compounds cannot be explained only by nonspecific iron chelation.

**Table 4:** Antimycobacterial activity of compounds **18**, **21**, and **22** against Mtb H37Ra in standard, iron‐enriched and iron‐depleted medium, expressed as minimum inhibitory concentration [µg mL^−1^].

	Normal medium		High Fe medium		Low Fe medium	
Cmpd	Repl 1	Repl 2	Repl 1	Repl 2	Repl 1	Repl 2
**18**	31.25	31.25	62.5	62.5	31.25	31.25
**21**	15.625	15.625	31.25	31.25	15.625	15.625
**22**	>500	>500	>500	>500	>500	>500
INH	0.125	0.125	0.125	0.125	0.125	0.125
RIF	0.003	0.003	0.006	0.006	0.002	0.001
CIP	0.25	0.25	0.25	0.25	0.25	0.25

INH—isoniazid, RIF—rifampicin, CIP—ciprofloxacin. Normal—Middlebrook 7H9/OADC/glycerol; high Fe—normal medium + 50µM FeCl_3_; low Fe—normal medium + 100µM 2,2′‐dipyridyl.

To find alternative explanations for the antimycobacterial activity of the reported compounds, we performed inhibition assays on selected, well‐documented mycobacterial enzymes and evaluated the pivotal compounds for effects on lipid and mycolic acid synthesis—see the following sections.

Regarding the relation of iron‐chelating properties to antiproliferative activity, there is a significant correlation for HepG2 cells. The antiproliferative activity increases in compounds **18** < **21** < **22** (Table 3 in Section 2.3.3), which is consistent with the relative iron‐chelating potency (Figure 4B). Based on this, the antiproliferative activity of the pivotal compounds can be linked to their iron‐chelating properties.

#### 2.4.2 Inhibition Assay — Enoyl‐[Acyl‐Carrier‐Protein] Reductase

**Figure 5:**
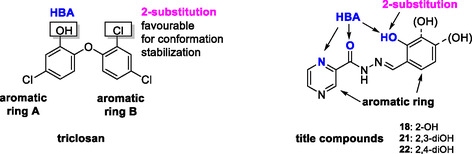
The enoyl‐ACP‐reductase inhibitor pharmacophore,^[^ ^25^ ^]^ depicted on a confirmed inhibitor triclosan and projected on the title benzylidene compounds.

The mycobacterial enoyl‐[acyl‐carrier‐protein] reductase (enoyl‐ACP reductase, InhA, UniProt accession: INHA_MYCTU, Uniprot ID: P9WGR1) is a clinically exploited antimycobacterial enzyme and the primary target of the first‐line antitubercular isoniazid (INH). Benzylidene derivatives **14**–**22** were tested for their inhibitory activity on InhA. The rationale for the testing is the presence of the InhA inhibition pharmacophore,^[^ ^25^ ^]^ exemplified on the inhibitor triclosan (TCL), in the title compounds (**Figure** **5**).

The inhibition assay was done following a procedure previously reported in the literature, using a confirmed inhibitor, 1‐(5‐((6‐bromopyridin‐2‐yl)amino)−1,3,4‐thiadiazol‐2‐yl)‐1‐(4‐methylthiazol‐2‐yl)ethan‐1‐ol, as a control.^[^ ^26^ ^]^ Compounds **18**, **21,** and **22**, as well as compounds with similarly substituted phenyl rings (**14**, **15**, **16**, **17**, and **20**), were tested for inhibition at 100 μM. The highest inhibition was obtained with **18** and **19**, the residual activity RA being **RA**_**18**_ **= 73%** and **RA**_**19**_ **= 77%,** respectively. Other tested compounds showed no relevant inhibition (RA ≥ 96%). Therefore, the reported (*E*)‐*N*’‐benzylidenepyrazine‐2‐carbohydrazide derivatives are not direct inhibitors of InhA. Full results and methodology are located in Supporting Information.

#### 2.4.3 Inhibition Assay — Isocitrate Lyase

**Figure 6:**
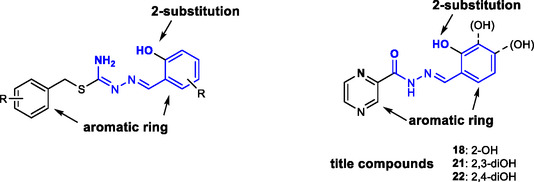
A comparison of the structure of reported isothiosemicarbazones^[^ ^29^ ^]^ (left) with the pivotal benzylidene compounds (right).

The mycobacterial isocitrate‐lyase (ICL, UniProt accession: ACEA_MYCTU, Uniprot ID: P9WKK7) is an enzyme responsible for the transformation of isocitrate into succinate and glyoxylate.^[^ ^27^ ^]^ It is essential for the survival of mycobacteria during host infection.^[^ ^28^ ^]^ A series of isothiosemicarbazones was previously studied for mycobacterial ICL inhibition and iron‐chelating properties.^[^ ^29^ ^]^ Two compounds, 5‐bromosalicylaldehyde‐*S*‐(4‐fluorobenzyl)‐isothiosemicarbazone and salicylaldehyde‐*S*‐(4‐bromobenzyl)‐isothiosemicarbazone, showed more potent ICL inhibition than 3‐nitropropionic acid (a standard inhibitor), along with good iron‐chelating properties.^[^ ^29^ ^]^ The rationale behind testing our title compounds (**18**, **21,** and **22**) for ICL inhibition is their structural similarity to the said isothiosemicarbazones (**Figure** **6**) and their shared ability to chelate iron ions.

The ICL inhibition assay was done following a procedure previously reported in the literature.^[^ ^29^ ^]^ 3‐Nitropropionic acid^[^ ^29^ ^]^ was used as a control. Compounds **18**, **21,** and **22** showed inhibition rates of 4.25%, 5.49%, and 3.22%, respectively, at 1 mM concentration. None of the additional compounds studied in the inhibition assay showed inhibition rates > 7%, with the highest inhibition obtained with compound **19** (6.56%). Therefore, inhibition of ICL is not the primary mechanism of action of the presented (*E*)‐*N’*‐benzylidenepyrazine‐2‐carbohydrazide derivatives. Full results and methodology are located in Supporting Information.

#### 2.4.4 The Effect on Mycolic Acids and Lipids Biosynthesis in Mycobacteria

**Figure 7:**
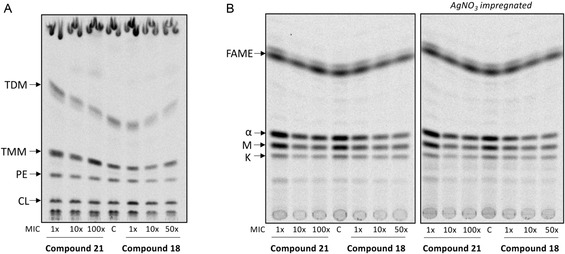
The effect of compounds **18** and **21** on biosynthesis of lipids (A) and mycolic acids (B) of Mtb H37Rv. A) The lipids were extracted from the cells cultivated in the presence of different concentrations of tested compounds and metabolically labeled with ^14^C acetate. Isolated lipids were separated in chloroform/methanol/water mixture (25/4/0.5) and visualized by autoradiography. B) Fatty acids were extracted from the cells cultivated as described above, derivatized to corresponding methyl esters, loaded on the standard (left) or AgNO_3_ impregnated (right) TLC plate, separated in *n*‐hexane/ethyl acetate (95:5; 3 runs), and visualized by autoradiography. TDM—trehalose dimycolates, TMM—trehalose monomycolates, PE—phosphatidylethanolamine, CL—cardiolipin. FAME—fatty acid methyl esters; *α*, M, K refer to alfa‐, methoxy‐, and keto‐mycolic acids methyl esters, respectively. C—untreated control.

To further test the originally expected connection between the observed antimycobacterial activity and inhibition of InhA, we probed the effects of compounds **18** and **21** on the biosynthesis of lipids and mycolic acids in mycobacteria. Specific changes in lipid/mycolic acids profile can be linked to inhibition of fatty acid synthase I (FAS‐I) system, any enzyme of the FAS‐II system (including InhA),^[^ ^30^ ^]^ but also other exploited and vulnerable targets in mycobacteria, such as trehalose monomycolate exporter (MmpL3),^[^ ^31^ ^]^ decaprenylphosphoryl‐*β*‐*D*‐ribofuranose 2′‐oxidase 1 and 2 (DprE1/2)^[^ ^32^ ^]^ or the folate pathway. Mtb H37Rv cells were treated with multiples of MIC of the tested compounds for 24 h in the presence of ^14^C acetate. The TLC analysis of ^14^C labeled lipids did not reveal any specific changes in the production of major phospholipids, but treatment with compound **21** led to a decrease of trehalose dimycolates with a parallel accumulation of trehalose monomycolates (**Figure** **7**A). This accumulation indicates impaired function of the MmpL3 transporter, which translocates trehalose monomycolates through the plasma membrane. Such phenotype was shown to be the result of direct inhibition of MmpL3 protein,^[^ ^31^ ^]^ or indirect membrane disruption.^[^ ^33^, ^34^ ^–^ ^35^ ^]^ TLC analysis of ^14^C labeled fatty/mycolic acids derivatized to corresponding methyl esters revealed no specific changes compared to untreated control, confirming no effect on FAS‐I, as well as FAS‐II systems (Figure 7B). Based on the obtained results, panel compounds with antimycobacterial activity **18** and **21** have little to no effect on lipid and mycolic acid biosynthesis.

### 2.5 Pharmacokinetic Properties

#### 2.5.1 Incubation of Compound 18 with Human Plasma

**Figure 8:**
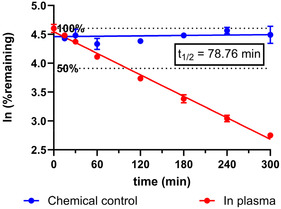
The time‐dependent concentration of parent compound **18** in human plasma.

Compound **18** was incubated with human plasma, and the concentrations of the parent compound were determined at specific time points from 0 to 300 min by HPLC–HRMS. The concentration of **18** declined with a half‐life of 79min (**Figure** **8**), but no notable increase in the intensity or concentration of any m/z values was observed in MS spectra, indicating no significant metabolites or degradation products of the parent compound (Figure S4, Supporting Information). Literature indicates that phenolic compounds are prone to interactions with plasma proteins, such as albumin, through hydrogen bonding and hydrophobic interactions.^[^ ^36^ ^]^ Studies have shown that phenolic compounds often form reversible noncovalent complexes with human serum albumin, influencing their pharmacokinetic profiles by reducing the concentration of free compound available in plasma.^[^ ^37^ ^]^ However, the observed half‐life of 79 min is much longer than expected for reversible protein binding, which occurs on a millisecond‐to‐second timescale. Moreover, protein precipitation with acetonitrile should release such reversibly bound compounds. Therefore, protein binding alone cannot explain the observed decrease, and other mechanisms such as slow degradation into undetectable products or irreversible binding to plasma components may also contribute.This understanding is crucial for elucidating the pharmacokinetic behavior of the compound and developing strategies to reduce nonspecific binding to enhance its therapeutic efficacy.

#### 2.5.2 Biotransformation of Compound 18 by Human Liver Microsomes (HLM)

Incubation with human liver microsomes (HLM) and further HPLC–HRMS analysis revealed no apparent Phase I metabolites for compound **18**. This absence of biotransformation can be explained by the already hydrophilic character of the studied compound (presence of ‐OH group, cLogP = 0.3).

## 3 Conclusion

To extend the knowledge of the biological activity of (*E*)‐*N*’‐benzylidenepyrazine‐2‐carbohydrazides and their derivatives, and to identify their possible mechanism of action, we synthesized a series of 33 compounds. The structure of compound **18** was confirmed through a crystallographic study, which verified an (*E*)‐configuration on the hydrazide linker. Substitution in position 2‐OH on the phenyl ring proved to be crucial for biological activity, as compounds **18** and **21** showed antimycobacterial activity (against Mtb H37Ra and H37Rv, *M. aurum*) and antifungal activity (against CA, CK, CT, LC, and TI). Compounds **18**, **21,** and **22** showed weak cytotoxic activity on five cancer cell lines *(*HepG2, A498, PC3, SKOV3, and U87 MG). The presence of the 2,4‐diOH substitution appears to eliminate antimycobacterial and antifungal activity while preserving cytotoxic activity. Compounds **18**, **21**, and **22** exhibited in vitro iron chelation activity comparable or superior to the reference chelator SIH. A correlation between iron chelation and antiproliferative activity supports a role for metal binding in the cytotoxic effects, whereas the antimycobacterial activity appears to involve additional or alternative mechanisms. Further evaluation of active compounds for effects on lipids and mycolic acids synthesis, enoyl‐ACP reductase inhibition, and ICL inhibition showed that these mechanisms are not responsible for the observed activity. Pharmacokinetic studies with compound **18** revealed no specific plasma metabolites, and no significant metabolites were detected after incubation with HLM.

In summary, this study provides new insights into the structure–activity relationships and potential mechanisms of (*E*)‐*N*’‐benzylidenepyrazine‐2‐carbohydrazides. The findings underscore the relevance of 2‐OH substitution for biological activity and support the hypothesis that iron chelation contributes to antiproliferative effects. These compounds may serve as starting points for further optimization, including prodrug strategies or combination therapies. Future work should focus on confirming the in vivo relevance of metal chelation and improving selectivity and pharmacokinetics for therapeutic development.

## 4 Experimental Section

4.1

4.1.1

##### General

All reagents and solvents (unless stated otherwise) were purchased from Merck (Schnelldorf, Germany) or Fluorochem (Hadfield, UK) and used without further purification. Reaction progress and purity of products were monitored using Silica 60 F_254_ TLC plates (Merck, Darmstadt, Germany). The NMR spectra were recorded on a Varian VNMR S500 (Varian, Palo Alto, CA, USA) at 500 MHz for ^1^H and 126 MHz for ^13^C or a Jeol JNM‐ECZ600R at 600 MHz for ^1^H and 151 MHz for ^13^C. The spectra were recorded in DMSO at ambient temperature. The chemical shifts reported as *δ* values in ppm are indirectly referenced to tetramethylsilane (TMS) via the solvent signal (2.49 for ^1^H and 39.7 for ^13^C). IR spectra were recorded on a NICOLET 6700 FT‐IR spectrophotometer (Nicolet, Madison, WI, USA) using the ATR‐Ge method. Elemental analysis was done on a Vario MICRO cube Element Analyzer (Elementar Analysensysteme, Hanau, Germany) with values given as a percentage. Compounds **7**–**9**, **18**, **21**, and **22** were analyzed by HPLC–HRMS using Dionex Ultimate 3000 UHPLC RS controlled by Chromeleon (version 7.2.9. build 11,323) software (Thermo Fisher Scientific, Germering, Germany) connected to Q Exactive Plus Orbitrap mass spectrometer with Thermo Xcalibur (version 3.1.66.10) software (Thermo Fisher Scientific, Bremen, Germany). Yields are stated in percentage and refer to the amount of pure product after all purification steps. Melting points were determined in an open capillary on a Stuart SMP30 melting point apparatus (Bibby Scientific Limited, Staffordshire, UK) and are uncorrected. Log*P* values were calculated using ChemDraw v21.1. (PerkinElmer Informatics, Waltham, MA, USA).

##### Chemistry: Pyrazine‐2‐Carbohydrazide (PCH)

Pyrazine‐2‐carboxamide (15 mmol, 1 eq) was dissolved in a 250 mL round‐bottom flask filled with THF (50 mL), and an excess of hydrazine was added (30% in H_2_O, 3 eq) and refluxed overnight. Then, the solvent was evaporated under reduced pressure on a rotary evaporator, and the solid leftover was charged with 50 mL of EtOH and refluxed overnight. The reaction was cooled down, and the precipitate was filtered off to yield the crude pyrazine‐2‐carbohydrazide (PCH), which was used for subsequent reactions without further purification.

##### Chemistry: General Procedure for Final Compounds **1**–**33**

Pyrazine‐2‐carbohydrazide (PCH, 2 mmol, 1 eq) was placed in a 100 mL round‐bottom flask with 30 mL of MeOH and heated to 70 °C, then the chosen aldehyde was added (2 mmol) in 10 mL of MeOH. After 3 h of stirring under reflux, the heating was turned off and a precipitate formed. The precipitate was filtered off and washed with cold MeOH to yield products of sufficient purity.

The full characterization and analytical data of intermediate **PCH** and final compounds **1–33** is available in Supporting Information.

##### Crystallography

For full description of methods used in crystallography, refer to Supporting Information.

##### Biological Methods: In Vitro Antimycobacterial Activity

Testing was performed by MABA and the results were expressed as MIC in µg mL^−1^ in comparison with isoniazid (INH), rifampicin (RIF), and ciprofloxacin (CIP) as standards. A full description of the methodology and characterization of the used strains is available in Supporting Information.

##### Biological Methods: In Vitro Antibacterial and Antifungal Activity

The microdilution broth method was performed according to EUCAST recommendations, with slight modifications. Antibacterial and antifungal activities were expressed as MIC. Internal quality standards, gentamicin (GEN), CIP, amphotericin B (AMB), and voriconazole (VRC) were included in the assays. A full description of the methodology and characterization of the used strains is available in Supporting Information.

##### Biological Methods: Cytotoxicity, Iron Chelation, Effect on Mycolic Acids and Lipids Biosynthesis, and Enzymatic Assays

For full description of the methods and additional results, refer to Supporting Information.

##### Biological Methods: Plasma Stability, Biotransformation by Human Liver Microsomes

For full description of the methods and additional results, refer to Supporting Information.

## Conflicts of Interest

The authors declare no conflicts of interest.

## Deposition Number

CCDC 2381295 (for compound **18**) contains the supplementary crystallographic data for this paper. These data are provided free of charge by the joint Cambridge Crystallographic Data Centre and Fachinformationszentrum Karlsruhe Access Structures service http://www.ccdc.cam.ac.uk/structures.

## Supporting information

Supplementary Material

## Data Availability

The data that support the findings of this study are available in the supplementary material of this article.
