# Supplementary material for: Investigating the Mechanism of Antimycobacterial and Antiproliferative Activity of (E)‐N’‐Benzylidenepyrazine‐2‐Carbohydrazides and their Derivatives
Source: ChemMedChem. 2025 Aug 29;20(18):e202500085. doi: 10.1002/cmdc.202500085 (PMC12479386; doi:10.1002/cmdc.202500085)
Supplement: Supplementary file 1 — Supplementary Material [file CMDC-20-e202500085-s001.pdf]

Investigating the mechanism of antimycobacterial and antiproliferative activity of (*E*)-*N*'-benzylidenepyrazine-2-carbohydrazides and their derivatives

Priam-Amedeo Houngbedji, Daria Elżbieta Nawrot, Ondřej Janďourek, Klára Konečná, Pavla Paterová, Pavel Bárta, Martina Hrast Rambaher, Eva Novotná, Carlo Castellano, Matteo Mori, Fiorella Meneghetti, Monika Záhorszká, Martin Novák, Jana Korduláková, Jan Zitko

SUPPORTING INFORMATION

1. Methods 3
  1.1 In vitro antimicrobial screening 3
    1.1.1 *In vitro* screening of antimycobacterial activity against *Mycobacterium tuberculosis* H37Ra, *M. smegmatis*, and *M. aurum* 3
    1.1.2 Determination of MIC in iron-enriched and iron-depleted medium 4
    1.1.3 *In vitro* screening of antimycobacterial activity against *Mycobacterium tuberculosis* H37Rv 4
    1.1.4 *In vitro* antibacterial evaluation 5
    1.1.5 *In vitro* antifungal evaluation 5
  1.2 *In vitro* cytotoxicity evaluation 7
  1.3 Crystallography 8
  1.4 Mechanism of action studies 9
    1.4.1 Iron chelation 9
    1.4.2 Enoyl-ACP reductase inhibition assay 9
    1.4.3 Isocitrate lyase inhibition assay 9
    1.4.4 Effect on lipids and mycolic acids synthesis 10
  1.5 HPLC purity and HRMS analysis of fluorinated compounds and selected compounds with biological activity 11
  1.6 *In vitro* stability testing in human plasma. 11
  1.7 Phase I metabolites identification *using* human liver microsomes (HLM) 12
2. Results 13
  2.1 Characterization and analytical data 14
  2.2 Representative NMR spectra (graphic) 22
    2.2.1 ^{1}H NMR and ^{13}C NMR of compound **18** 22
    2.2.2 ^{1}H NMR and ^{13}C NMR of compound **21** 23
    2.2.3 ^{1}H NMR and ^{13}C NMR of compound **22** 24
  2.3 HPLC-HMRS analysis of fluorinated compounds and selected compounds with biological activity…. 25
  2.4 Crystallography of compound **18** 31
  2.5 Results of incubation of compound **18** with human plasma 33

2.6 Results of *in vitro* antimicrobial screening 34
2.6.1 Antimycobacterial activity 34
2.6.2 Antibacterial activity 35
2.6.3 Antifungal activity 36
2.7 Mechanism of action studies 37
2.7.1 Results of enoyl-ACP reductase (InhA) inhibition assay 37
2.7.2 Results of isocitrate lyase (ICL) inhibition assay 38
3. References 39

1. Methods

1.1 In vitro antimicrobial screening

1.1.1 *In vitro* screening of antimycobacterial activity against *Mycobacterium tuberculosis* H37Ra, *M. smegmatis*, and *M. aurum*

The antimycobacterial assay was performed with rapidly growing *Mycolicibacterium smegmatis* DSM 43465 (ATCC 607) and *Mycolicibacterium aurum* DSM 43999 (ATCC 23366) obtained from the German Collection of Microorganisms and Cell Cultures (Braunschweig, Germany). The avirulent strain of *Mycobacterium tuberculosis* H37Ra ITM-M006710 (ATCC 9431) was obtained from Belgian Co-ordinated Collections of Micro-organisms (Antwerp, Belgium). The technique used for activity determination was the microdilution broth panel method using 96-well microtitration plates. The culture medium was Middlebrook 7H9 broth (Merck, Darmstadt, Germany) enriched with 0.4% glycerol (Merck, Darmstadt, Germany) and 10% Middlebrook OADC growth supplement (Himedia, Mumbai, India). The mycobacterial strains were cultured on supplemented Middlebrook 7H9 agar and suspensions were prepared in supplemented Middlebrook 7H9 broth. The final density was adjusted to 1.0 on the McFarland scale and diluted in the ratio of either 1:20 (for rapidly growing mycobacteria) or 1:10 (for slow-growing mycobacteria) with broth. The tested compounds were dissolved in DMSO (Merck, Darmstadt, Germany), then Middlebrook broth was added to obtain a concentration of 2000 µg/mL. The standards used for activity determination were isoniazid (INH), rifampicin (RIF), and ciprofloxacin (CIP) (Merck, Darmstadt, Germany). Final concentrations were reached by binary dilution and addition of mycobacterial suspension and were set as 500, 250, 125, 62.5, 31.25, 15.625, 7.81, and 3.91 µg/mL. Isoniazid was diluted in the range 500–3.91 µg/mL for screening against rapidly growing mycobacteria, and 1-0.0078 µg/mL for *M. tuberculosis*. Rifampicin final concentrations ranged from 50 to 0.39 µg/mL for rapidly growing mycobacteria, and from 0.1 to 0.00078 µg/mL for *M. tuberculosis*. Ciprofloxacin was used for screening the antimycobacterial activity with final concentrations of 1, 0.5, 0.25, 0.125, 0.0625, 0.0313, 0.0156, 0.0078 µg/mL. The final concentration of DMSO did not exceed 2.5% (v/v) and did not affect the growth of all strains. Positive (broth, DMSO, bacteria) and negative (broth, DMSO) growth controls were included. Plates containing slow-growing mycobacteria were sealed with polyester adhesive film and incubated in the dark at 37 °C without agitation. A 0.01% solution of resazurin sodium salt was added after 48 hours of incubation for *M. smegmatis*, 72 hours for M. aurum, and 120 hours for *M. tuberculosis*. Microtitration panels were then incubated for addiitonal 2.5 hours to determine the activity against *M. smegmatis*, 4 hours for *M. aurum,* and 18 hours for *M. tuberculosis*. The antimycobacterial activity was expressed as minimal inhibition concentration (MIC), and the value was read based on stain colour change (blue colour – active compound; pink colour – inactive compound). All experiments were conducted in duplicates.

1.1.2 Determination of MIC in iron-enriched and iron-depleted medium

To assess the dependence of MIC on iron concentration in the cultivation medium, the standard assay was repeated with M. tuberculosis H37Ra using standard, iron-enriched, and iron-depleted media. Mycobacteria were cultivated in standard Middlebrook 7H9 broth supplemented with OADC and glycerol. After incubation, the suspension was centrifuged, the supernatant was discarded, and working suspensions were prepared in the respective media. The first medium consisted of Middlebrook 7H9 broth supplemented with OADC and glycerol (standard conditions). The second medium was enriched with iron: after preparation of the standard medium, a sterile aqueous solution of $FeCl_3$ was added to reach a final concentration of 50 µM. The third medium, mimicking iron-depleted conditions, was prepared analogously by adding a sterile solution of 2,2′-dipyridyl in DMSO to the standard medium, yielding a final concentration of 100 µM. The final DMSO concentration was 0.1%, which did not affect the growth of mycobacteria.

Positive growth controls were included in all tested media to exclude any potential inhibitory effects of the additives on mycobacterial growth. All experiments were conducted in duplicates.

1.1.3 *In vitro* screening of antimycobacterial activity against *Mycobacterium tuberculosis* H37Rv

Testing was performed according to the previously published method.[1] The tested strain *Mycobacterium tuberculosis* H37Rv CNCTC My 331/88 (ATCC 27294) was obtained from the Czech National Collection of Type Cultures (CNCTC), National Institute of Public Health (Prague, Czech Republic). Middlebrook 7H9 broth of declared pH = 6.6 (Sigma-Aldrich) enriched with 0.4% of glycerol (Sigma-Aldrich) and 10% of OADC growth supplement (oleic acid, albumin, dextrose, catalase; Himedia, Mumbai, India) was used for cultivation. The tested compounds were dissolved and diluted in DMSO and mixed with broth (25 µL of DMSO solution in 4.475 mL of broth) and then placed (100 µL) into microplate wells. Mycobacterial inocula were suspended in isotonic saline solution, and the density was adjusted to 0.5–1.0 on the McFarland scale. These suspensions were diluted by 10-1 and used to inoculate the testing wells, by adding 100 µL of mycobacterial suspension per well. The final concentrations of tested compounds in wells were 100, 50, 25, 12.5, 6.25, 3.13, and 1.56 µg/mL. INH was used as a positive control (inhibition of growth). The negative control (visible growth) consisted of broth plus mycobacterial suspension plus DMSO. A total of 30 µL of Alamar Blue working solution (1:1 mixture of 0.01% resazurin sodium salt (aq. sol.) and 10% Tween 80) was added after five days of incubation. Results were determined after 24 h of incubation. The MIC (in µg/mL) was determined as the lowest concentration that prevented the blue-to-pink colour change. The MIC values of INH were 0.1–0.2 µg/mL against. All experiments were conducted in duplicates.

1.1.4 *In vitro* antibacterial evaluation

Microdilution broth method according to The European Committee on Antimicrobial Susceptibility Testing (EUCAST) recommendations, with slight modifications, was employed for antibacterial activity evaluation *in vitro*.[2] Eight bacterial strains were included in the study for the screening of antibacterial activity, namely *Staphylococcus aureus* subsp. *aureus* ATCC 29213 (CCM 4223); *Staphylococcus aureus* subsp*. aureus,* methicillin-resistant, ATCC 43300 (CCM 4750); *Staphylococcus epidermidis* ATCC 12228 (CCM 4418); *Enterococcus faecalis* ATCC 29212 (CCM 4224); *Escherichia coli* ATCC 25922 (CCM 3954); *Klebsiella pneumoniae* ATCC 10031 (CCM 4415); *Acinetobacter baumannii* ATCC 19606 (DSM 30007); *Pseudomonas aeruginosa* ATCC 27853 (CCM 3955). Strains were purchased from the Czech Collection of Microorganisms (CCM, Brno, Czech Republic) or from the German Collection of Microorganisms and Cell Cultures (DSM, Braunschweig, Germany). The technique used for activity determination was based on a microdilution broth panel method using 96-well microtiter plates. The cultivation was done in Cation-adjusted Mueller-Hinton broth (CAMHB, M-H 2 Broth, Sigma-Aldrich), buffered to pH 7.0 at 35±2 °C. Tested compounds were dissolved in DMSO to produce stock sample solutions. The final concentration of DMSO in the testing medium did not exceed 1% (v/v) of the total sample solution composition. Positive growth controls (microbes without exposure to tested compound in cultivation medium), negative growth controls (cultivation medium only), and the internal quality standards, gentamicin (GEN) and ciprofloxacin (CIP) were included in assays. Antibacterial activity was evaluated after 24 and 48h of static incubation at 35±2 °C by visual inspection and expressed as minimum inhibitory concentration (MIC). See Table S1 for standards.

Table S1. MIC values of standards used in antibacterial activity evaluation assay expressed in µg/mL

Standard	SA	MRSA	SE	EF	EC	KP	ACI	PA
	24h	24h	24h	24h	24h	24h	24h	24h
**CIP**	0.256	0.128	0.128	1.024	0.008	0.008	0.256	0.512
**GEN**	0.5	>8	0.125	>8	1	0.5	8	0.512

GEN – gentamicin, CIP - ciprofloxacin

1.1.5 *In vitro* antifungal evaluation

Microdilution broth method according to EUCAST recommendations, with slight modifications, was employed for antifungal activity screening *in vitro*.[3-4] Four yeast reference strains*, Candida albicans* ATCC 24433 (CCM 8320); *Candida krusei* ATCC 6258 (CCM 8271); *Candida parapsilosis* ATCC 22019 (CCM 8260); *Candida tropicalis* ATCC 750 (CCM 8264), and four filamentous fungi, *Aspergillus fumigatus* ATCC 204305; *Aspergillus flavus* CCM 8363; *Lichtheimia corymbifera* CCM 8077; and *Trichophyton interdigitale* ATCC 9533 (CCM 8377), purchased from the Czech Collection of Microorganisms (CCM, Brno, Czech Republic) or from the American Type Collection Cultures (ATCC, Manassas, VA, USA), were included in antifungal activity evaluation *in vitro*. The technique used for activity determination was based on a microdilution broth panel method with 96-well microtiter plates. The cultivation was done in RPMI-1640 medium, with glutamine and

2% glucose, buffered to pH 7.0 with MOPS (3-(*N*-morpholino)propanesulfonic acid). Tested compounds were dissolved in DMSO to produce stock sample solutions. The final concentration of DMSO in the testing medium did not exceed 1% (v/v) of the total sample solution composition. Positive growth controls (microbes without exposure to tested compound in cultivation medium), negative growth controls (cultivation medium only), and the internal quality standards, amphotericin B (AMB) and voriconazole (VRC), were included in assays. Static incubation was performed in the dark and in a humid atmosphere, at 35±2 °C, for 24 and 48h (72 and 120h for *Trichophyton interdigitale*, respectively). The antifungal activity of standards was evaluated after visual inspection and expressed as MIC, reported in µM, see Table S2 for standards.

Table S2. MIC values of standards used in antifungal activity evaluation assay expressed in µg/mL

Standard	CA	CK	CP	CT	AF	Afla	LC	TI
	24h	24h	24h	24h	48h	48h	48h	120h
**AMB**	0.5	0.5	0.5	1	1	4	0.125	1
**VRC**	>16	16	>16	>16	1	1	>16	>16

AMB - amphotericin B, VRC - voriconazole

1.2 *In vitro* cytotoxicity evaluation

All used cell lines are commercially available from American Type Culture Collection (ATCC) and were purchased less than 10 years ago. Cells were tested on mycoplasma contamination upon their delivery. Human hepatocellular carcinoma cells HepG2 (ATCC HB 8065) and human epithelial glioblastoma cells U 87 MG (ATCC HBT 14) were cultured in Dulbecco's Modified Eagles Medium High Glucose (denoted DMEM High Glucose) supplemented with fetal bovine serum (10%), Non-Essential Amino Acids (5%), and penicillin/streptomycin (5%). Human prostate adenocarcinoma cells PC-3 (ATCC CRL-1435) were cultured in Kaighn's Modification of Ham's F-12 Medium (denoted F-12K Medium; Gibco, ThermoFisher Scientific USA) supplemented with fetal bovine serum (10%) and penicillin/streptomycin (5%). Human epithelial kidney carcinoma cells A498 (ATCC HBT 44) were cultured in Eagle's Minimum Essential Medium (denoted EMEM) supplemented with fetal bovine serum (10%), L-glutamine and penicillin/streptomycin (5%). Human ovary adenocarcinoma cells SK OV 3 (ATCC HTB 77) were cultured in McCoy's 5A Medium (Biosera, France) supplemented with fetal bovine serum (10%), L-glutamine (5%), pyruvate (5%), Non-Essential Amino Acids (5%) and penicillin/streptomycin (5%). All media and supplements were purchased from Merck (USA) unless otherwise stated.

All cell lines were cultured at 37 °C, under 5% CO_2 humidified atmosphere and grown to confluence. Before the experimental day, cells were seeded into a 96-well plate with the initial cell number of 1.0×10^4 in 100 µL of cell media per well. After the 24h incubation at 37 °C, cells were treated with the tested compounds in the final volume of 100 µL containing 1% of DMSO (v/v). Cells were incubated with the compounds at the concentrations as follows 1, 5, 10, 25, 50, 100, 250, 500, and 1000 µM for 24h. Controls were treated with appropriate cell culture medium only containing 1% of DMSO (v/v) at 37 °C for 24h. The absolute mortality of cells was also analysed with 10% DMSO (v/v) dissolved in appropriate cell culture medium at 37 °C for 24h. Controls and compound concentrations were made in triplicate of wells.

When the 24h incubation was over, the reagent from the kit CellTiter 96 AQueous One Solution Cell Proliferation Assay (CellTiter 96; PROMEGA, Fitchburg, USA) was added. After the 2h incubation at 37 °C, absorbance in each sample/control well was recorded at 490 nm (TECAN, Infinita M200, Austria).

The 50% inhibitory concentrations (IC_{50}) values were determined from the dose-response curves in GraphPad Prism 10 (GraphPad Software, Boston, MA USA). Viability (%) was plotted as a function of concentration (log values), fitted to a sigmoidal curve and based on this curve, the half maximal inhibitory concentration value was determined representing the concentration of a compound required for 50% inhibition.

1.3 Crystallography

Colorless, prismatic crystals of **18** were obtained at room temperature by the slow evaporation of an acetonitrile solution. X-ray intensity data were collected with a Bruker Apex II CCD three-circle diffractometer (Bruker, Billerica, MA, USA), working at room temperature with graphite monochromatized Mo-Kα X-radiation (λ = 0.71073 Å). A total of 12,032 Bragg reflections were collected in the range 2-23°, using omega scans with a step size of 0.5° and an exposure time of 50 s/frame. A least-squares refinement of the positions of 3245 independent reflections with I > 10σ(I) was applied to determine accurate unit cell dimensions, revealing a metrically monoclinic unit. The analysis of the systematic absences indicated the space group $P2_1/c$. The intensity data were processed using SAINT[5] for integration, as well as background and Lorentz-polarization corrections. Absorption effects were empirically evaluated with SADABS[6], and the resulting corrections were applied to the data. The structure was solved by direct methods using SIR2019/3[7] and completed by iterative cycles of full-matrix least squares refinement on F_o^2 and ΔF synthesis using SHELXL-2019/3[8] within the WinGX suite (WinGX v.2023.1).[9] Hydrogen atoms were introduced at calculated positions in their described geometries and allowed to ride on the attached atom with fixed, standard isotropic thermal parameters. The structure was analyzed with PARST,[10] and the graphical representations were rendered with Mercury 2024.1.0.[11] Hirshfeld surface (HS) analysis was performed with CrystalExplorer21,[12] and enrichment ratios were computed with MoProViewer 1.2000.[13]

Crystal data for 18. Formula: $C_{24}H_{20}N_8O_4$ (ASU); MW: 484.48 g/mol (ASU); Temperature: 293(2) K; Wavelength: 0.71073 Å; Crystal system: monoclinic; Space group: $P2_1/c$; Unit cell dimensions: a = 8.0746(16) Å, b = 21.895(4) Å, c = 12.711(3) Å, β = 93.03(3)°; V = 2244.0(8) Å^3; Z = 4; D_{calc} = 1.434 Mg/m^3; Absorption coefficient: 0.103 mm^{-1}; F(000): 1008; Crystal size: 0.06 × 0.03 × 0.02 mm; $2\theta_{min}$ = 1.854°; $2\theta_{max}$ = 23.076°; Limiting indices = $-8 \le h \le 8$, $-24 \le k \le 24$, $-14 \le l \le 13$; Collected reflections: 12,032; Independent reflections: 3136 (R_{int} = 0.0976); Completeness to 23.076°: 99.9%; Refinement method: Full-matrix least-squares on F^2; Data/restraints/parameters: 3136/0/331; GOOF: 0.794; Final R indices [I > 2σ(I)]: R1 = 0.0538, wR2 = 0.1240; R indices (all data): R1 = 0.1207, wR2 = 0.1444; Largest diff. peak and hole: 0.436 and -0.207 e·Å^{-3}. CCDC deposition number: 2381295.

1.4 Mechanism of action studies

1.4.1 Iron chelation

The iron chelation study was performed following a previously reported procedure.[14]

1.4.2 Enoyl-ACP reductase inhibition assay

Enoyl-[acyl-carrier-protein] reductase (enoyl-ACP reductase, InhA, UniProt accession: INHA_MYCTU, Uniprot ID: P9WGR1) from *Mycobacterium tuberculosis* was overexpressed and purified according to previously described methods.[15] The enzymatic activity of InhA was assessed spectrophotometrically by monitoring NADH oxidation at 340 nm. Kinetic assays were conducted at 25 °C over a duration of 10 minutes. Stock solutions of all compounds (10 mM) were prepared in DMSO, with the final concentration of DMSO in the assay mixture kept at 1% v/v. Reactions were initiated by adding InhA (50 nM) to solutions containing the inhibitor (100 µM), 2-trans-dodecenoyl-coenzyme A (DD-CoA, 50 µM), and NADH (100 µM) in 30 mM PIPES buffer at pH 6.8. All compounds were soluble under the assay conditions. Control reactions were performed under the same conditions without the inhibitor, using 1% v/v DMSO. To rule out potential non-specific (promiscuous) inhibition, all compounds were additionally tested in the presence of Triton X-114 (0.005%). The inhibitory activity of each compound was expressed as a percentage of InhA activity inhibition, based on the initial reaction velocity relative to the control without the inhibitor. All inhibition assays were carried out in triplicates, with standard deviations within ±10%.

1.4.3 Isocitrate lyase inhibition assay

Mycobacterial isocitrate lyase (ICL, UniProt accession: ACEA_MYCTU, Uniprot ID: P9WKK7) was prepared as previously described[16] with some modifications in protein expression and purification. *Escherichia coli* BL21(DE3) cells transformed with the plasmid pET-28b(+) encoding the ICL were grown at 37 °C in Luria-Bertani (LB) medium containing kanamycin (50 µg/ml) to an optical density of OD_{595nm} = 0.6. Protein expression was induced by the addition of isopropyl β-D-1-thiogalactopyranoside (IPTG, 0.5 mM final concentration) and the cell culture was further incubated at 30 °C for 8 hours. Cells were harvested by centrifugation (10,000 × g for 10 min at 4 °C), the pellet was resuspended in BugBuster Protein Extraction Reagent (Novagen) containing Benzonase Nuclease and incubated for 20 min on a shaking platform at room temperature according to the manufacturer's protocol. The histidine-tagged protein was purified from the cytosol using the NGC Chromatography System (Bio-Rad) equipped with a 1 mL HisTrap FF column (Cytiva, Uppsala, Sweden). First, the cell lysate was centrifuged (16,000 × g for 20 min at 4 °C) and the insoluble cell debris was removed. The supernatant was adjusted to contain 30 mM imidazole, loaded onto the column and washed with the buffer A containing 20 mM Tris-HCl, 150 mM NaCl, 20% glycerol (v/v) and 30 mM imidazole (pH 7.4). Pure recombinant protein was then eluted by using increasing concentrations of buffer B

(20 mM Tris-HCl, 150 mM NaCl, 20 % glycerol (v/v) and 500 mM imidazole (pH 7.4)). The fractions of purified protein were pooled, buffer B was exchanged for 20 mM K phosphate buffer (pH 7.4) using Amicon Ultra-4 Centrifugal Filter Units (Merck Millipore Ltd.) and supplemented with glycerol (final concentration 20%). The purity of the protein was verified by SDS-polyacrylamide gel electrophoresis. The Bradford method[17] was used to determine the concentration of the protein.

To investigate the effect of the compounds on ICL activity, 176 µL of reaction buffer (50 mM potassium phosphate, 4 mM magnesium chloride, 12 mM L-cysteine, 4 mM phenylhydrazine hydrochloride, pH 7.0) and recombinant enzyme (approximately 0.2 µg) were pipetted into the wells of 96-well UV-transparent plates (Thermo). Subsequently, 4 µl of 0.5 mM compounds, DMSO (vehiculum), 3-nitropropionic acid (positive control) or water were added. After pre-incubation for 10 minutes at 37 °C, the reactions were started by the addition of 20 µL of (+)-potassium Ds-*threo*-isocitrate (2 mM final concentration in the reaction). The cleavage of the substrate associated with the formation of glyoxylic acid phenylhydrazone[18] was continuously monitored using a Tecan Infinite M200 plate reader (Schoeller) set to 324 nm and 37 °C.

The compounds were dissolved in DMSO, 3-nitropropionic acid (a positive control) was dissolved in demineralised water and screened on the same plates as the tested compounds.

1.4.4 Effect on lipids and mycolic acids synthesis

Culture of *M. tuberculosis* H37Rv was grown in Middlebrook 7H9 broth supplemented with 10% albumin-dextrose-catalase and 0.05% Tween 80, shaking at 120 RPM at 37 °C, until OD_{600} 0.18. Cultures (95 µL) were added into Eppendorf tubes containing 2 µL of DMSO or 2 µL of tested compounds **21** and **18** at final concentrations relative to their MIC: 1x, 10x or 100x/50x the MIC. At the same time [^{14}C]-acetate (ARC; specific activity 110 mCi/mmol) was added in final concentration 0.5 µCi/mL and cultures were incubated at 37 °C under static conditions for 24 h.

Lipids were extracted from 100 µL culture aliquots with 1.5 mL chloroform: methanol (2: 1) at 65 °C for 3 h. After extraction, 150 µL of ddWater was added, the samples were mixed, centrifuged at 3 000 x g for 3 min and lower organic phase was removed and dried under nitrogen. Extracted lipids were washed with chloroform: methanol: ddWater (4: 2: 1), dried under nitrogen, resuspended in 50 µL chloroform: methanol (2: 1) and 5 µL were loaded on silica gel 60 F_{254} plates (Merck). A mixture of chloroform: methanol: ddWater (20 : 4 : 0.5) was used as the eluent. ^{14}C-labeled lipids were visualized using an Amersham™ Typhoon™ Biomolecular Imager.

For mycolic acids extraction, 100 µL cultures were treated with 1 mL of tetrabuthylammonium hydroxide (15%) and incubated at 100 °C overnight. Samples were methylated by adding 1.5 mL of dichloromethane, 1 mL of ddWater, and 150 µL of iodomethane, and incubated at room temperature under rotation for 4 h. Methylated samples were washed twice with ddWater, and organic phases were dried under nitrogen. Fatty and mycolic acid methyl esters (FAME and MAME) were extracted with 2 mL of diethyl ether, dried, and resuspended in 50 µL chloroform: methanol (2: 1) and 5 µL were loaded on TLC plate, which was developed

in *n*-hexane: ethyl acetate (95: 5; 3 runs). Unsaturated forms of MAME were analyzed on silica plates impregnated with 5% $AgNO_3$. ^{14}C-labeled FAME/MAME were visualized as described above.

1.5 HPLC purity and HRMS analysis of fluorinated compounds and selected compounds with biological activity

The HPLC system used in this study was Dionex Ultimate 3000 UHPLC RS consisting of RS Pump, RS Column Compartment, RS Autosampler and Diode Array Detector controlled by Chromeleon (version 7.2.9. build 11323) software (Thermo Fisher Scientific, Germering, Germany) connected to Q Exactive Plus Orbitrap mass spectrometer with Thermo Xcalibur (version 3.1.66.10.) software (Thermo Fisher Scientific, Bremen, Germany).

The synthesized compounds were analyzed by LC-MS system to determine their purity and obtain high-resolution mass spectra. A reverse-phase C18 column Kinetex EVO (50 x 2.1 mm, 1.7 µm, Phenomenex, Torrance, CA, USA) was used as a stationary phase, and purified water with 0.1% formic acid (mobile phase A) and LC-MS grade acetonitrile with 0.1% formic acid (mobile phase B) were used as the mobile phases. Gradient elution was used to determine purities and mass spectra. The method started with 5% B for 0.3 min, then the gradient switched to 100% B in the third min, remained at 100% B for 0.7 min and then went back to 5% B with equilibration for 3.5 min. The total run time of the method was 7.5 min. The column temperature was kept constant at 27 °C, the flow of the mobile phase was 0.4 mL/min, and the injection volume was 1 µL. Detection was performed by UV detector (λ=254 nm) and by mass spectrometry in positive mode. HRMS spectra were collected from the total ion current in the scan range 105–1000 m/z, with the resolution set to 140,000. Settings of the heated electrospray source were: spray voltage 3.5 kV; capillary temperature 220 °C; sheath gas 55 arbitrary units; auxiliary gas 15 arbitrary units; spare gas 3 arbitrary units; probe heater temperature 220 °C; max spray current 100 mA and S-lens RF Level 50. Solvents and other common chemicals were purchased from VWR (Stribrna Skalice, Czech Republic). Solvents for chromatographic procedures were supplied in LC-MS grade.

1.6 *In vitro* stability testing in human plasma.

Compound **18** was incubated with Human Pooled Plasma (Batch S00G71, Biowest, France). Briefly, compound 18 was dissolved in DMSO to produce stock sample solutions. 5 uL of stock solution was added to 1995 uL of human plasma to initiate the reaction. The final concentration of DMSO in the incubation mixture did not exceed 0.5% (v/v) and the concentration of compound 18 was set at 1 µM. Compound was incubated for 0, 15, 30, 60, 120, 180, 240 and 300 min at 37 °C. The reactions were stopped by transferring 100 µL of incubate to 300 µL of acetonitrile containing internal standard [IS; compound **67**[19]] at the appropriate time points and centrifuged at 12,000 rpm for 5 min at 4 °C to precipitate the protein. After that, 150 µL of

supernatant was transferred to the vial and analyzed by LC-MS under the same conditions as in the section HPLC purity and HRMS analysis. The areas of the compounds (Acmp) and internal standards (AIS) were detected in extracted ion chromatograms from the mass spectrometer data in positive mode.

1.7 Phase I metabolites identification *using* human liver microsomes (HLM)

The methodology for the identification of phase I metabolites was inspired by Novak *et al*.[20] Briefly, 25 µL of pooled Human Liver Microsomes (concentration 0.5 mg/mL, H2620, LOT no. 1210347, SekiSui, XenoTech, Canada) was mixed with 5 µL of 30 µM stock solution of tested compound 18 and 420 µL of 0.1M potassium phosphate buffer solution (pH = 7.4, adjusted by addition of KOH) and preincubated for 5 min (300 rpm, 37 °C). The biotransformation reaction was started with the addition of 50 µL of RapidStart NADPH Regenerating System (K5000, LOT. 1910008, SekiSui, XenoTech, Canada) and then incubated for 3 hours. The reaction was terminated by the addition of 500 µL of cooled acetonitrile (-20 °C) with 1 µM internal standard [IS, compound 67[19]] and centrifuged for 5 min (14 000 rpm, 20 °C). After that, 800 µL of the supernatant was evaporated to dryness and stored in a freezer (-80 °C). Prior to HPLC-MS analysis, all samples were thawed at laboratory temperature and then reconstituted in 100 µL of 50% acetonitrile in water (v/v). Three types of blank samples were prepared in the same way, with a one-step exception. In the biological blank sample, 5 µL of DMSO was added instead of 5 µL stock solution; 50 µL of water instead of HLM was added in the chemical blanks, and in the case of the control blank sample, 25 µL of water was added instead of 25 µL of RapidStart System.

The identification of phase I metabolites was obtained by the HPLC-HRMS system mentioned above. In this study, a reverse phase method was used on a C18 column (Kinetex C18, 3.0 x 150 mm, 2.6 µm, Phenomenex, Torrance, California, USA). The mobile phase was as above: water and acetonitrile with formic acid. Initially, 5% B flowed for 1 min, and then the composition increased to 100% B in 14 min. After 1 min of the constant flow of 100% B, the composition of mobile phases reverted to 5% B and equilibrated for 3 min. The total run time of the method was 18 min. The column temperature was set to 27 °C and the injection volume was 5 µL. The detection parameters were set to the same as above. The metabolites were determined in total ion current spectra from the mass spectrometry data obtained in positive mode.

2. Results

Table S3. Synthesized compounds with SMILES and calculated logP (ChemDraw v21.1)

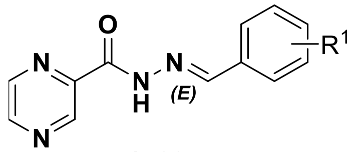

1–23

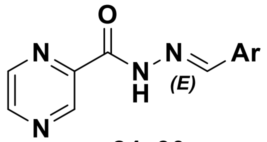

24–30

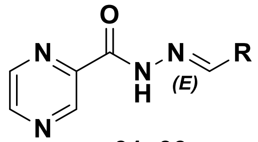

31–33

CODE	R[1]	log*P*[a]	SMILES
1	2-Cl	1.67	O=C(N/N=C/c1ccccc1Cl)c2cnccn2
2	3-Cl	1.67	O=C(N/N=C/c1cccc(Cl)c1)c2cnccn2
3	4-Cl	1.67	O=C(N/N=C/c1ccc(Cl)cc1)c2cnccn2
4	2-Br	1.94	O=C(N/N=C/c1ccccc1Br)c2cnccn2
5	3-Br	1.94	O=C(N/N=C/c1cccc(Br)c1)c2cnccn2
6	4-Br	1.94	O=C(N/N=C/c1ccc(Br)cc1)c2cnccn2
7	2-F	1.27	O=C(N/N=C/c1ccccc1F)c2cnccn2
8	3-F	1.27	O=C(N/N=C/c1cccc(F)c1)c2cnccn2
9	4-F	1.27	O=C(N/N=C/c1ccc(F)cc1)c2cnccn2
10	2,3,6-triCl	2.79	O=C(N/N=C/c1c(Cl)ccc(Cl)c1Cl)c2cnccn2
11	2-NO_2	0.52	O=C(N/N=C/c1ccccc1[N+](=O)[O-])c2cnccn2
12	3-NO_2	0.52	O=C(N/N=C/c1cccc([N+](=O)[O-])c1)c2cnccn2
13	4-NO_2	0.52	O=C(N/N=C/c1ccc([N+](=O)[O-])cc1)c2cnccn2
14	2-OMe	0.99	COc1ccccc1/C=N/NC(=O)c2cnccn2
15	4-OH, 3-OMe	0.60	COc2cc(/C=N/NC(=O)c1cnccn1)ccc2O
16	3-OMe	0.99	COc2cccc(/C=N/NC(=O)c1cnccn1)c2
17	4-OMe	0.99	COc2ccc(/C=N/NC(=O)c1cnccn1)cc2
18	2-OH	0.72	O=C(N/N=C/c1ccccc1O)c2cnccn2
19	3-OH	0.99	O=C(N/N=C/c1cccc(O)c1)c2cnccn2
20	4-OH	0.72	O=C(N/N=C/c1ccc(O)cc1)c2cnccn2
21	2,3-diOH	0.33	O=C(N/N=C/c1cccc(O)c1O)c2cnccn2
22	2,4-diOH	0.33	O=C(N/N=C/c1ccc(O)cc1O)c2cnccn2
23	4-OH, 3-NO_2	0.13	O=C(N/N=C/c1ccc(O)c([N+](=O)[O-])c1)c2cnccn2
**CODE**	**Ar (heteroaromate)**	**log*P*[a]**	**SMILES**
24	pyridin-2-yl	0.22	O=C(N/N=C/c1ccccn1)c2cnccn2
25	pyridin-3-yl	0.22	O=C(N/N=C/c1cccnc1)c2cnccn2
26	pyridin-4-yl	0.22	O=C(N/N=C/c1ccncc1)c2cnccn2
27	furan-2-yl	0.27	O=C(N/N=C/c1ccco1)c2cnccn2
28	aminothiazol-2-yl	0.50	O=C(N/N=C/c1nccs1)c2cnccn2
29	thiophen-2-yl	1.10	O=C(N/N=C/c1cccs1)c2cnccn2
30	3-methylthiophen-2-yl	1.43	Cc1ccsc1/C=N/NC(=O)c2cnccn2
**CODE**	**R**	**log*P*[a]**	**SMILES**
31	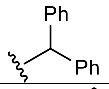	2.58	O=C(N/N=C/C(c1ccccc1)c2ccccc2)c3cnccn3
32	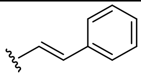	1.00	O=C(N/N=C/C=C/c1ccccc1)c2cnccn2
33	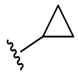	0.52	O=C(N/N=C/C=C/C1CC1)c2cnccn2

2.1 Characterization and analytical data

Pyrazine-2-carbohydrazide (PCH)

White solid, yield 70%; ^{1}H NMR (600 MHz, DMSO-d_6) δ 10.08 (s, 1H), 9.09 (d, *J* = 1.4 Hz, 1H), 8.79 (d, *J* = 2.5 Hz, 1H), 8.65 (dd, *J* = 2.6, 1.5 Hz, 1H), 4.64 (s, 2H); ^{13}C NMR (126 MHz, DMSO-d_6) δ 163.43, 155.99, 148.18, 133.53, 123.57; Melting point: 169–170 °C; Elemental analysis calculated for $C_5H_6N_4O$ (MW 138.13): 43.48% C, 4.38% H, 40.56% N, measured: 43.39% C, 4.22% H, 40.56% N; CAS Registry Number: 768-05-8

(*E*)-*N*'-(2-chlorobenzylidene)pyrazine-2-carbohydrazide (1)

White solid, yield 88%; ^{1}H NMR (500 MHz, DMSO-d_6) δ 12.60 (s, 1H), 9.27 (d, *J* = 1.5 Hz, 1H), 9.09 (s, 1H), 8.93 (d, *J* = 2.5 Hz, 1H), 8.79 (dd, *J* = 2.5, 1.5 Hz, 1H), 8.08–8.00 (m, 1H), 7.57–7.49 (m, 1H), 7.51–7.35 (m, 2H); ^{13}C NMR (126 MHz, DMSO-d_6) δ 159.97, 148.07, 146.05, 144.72, 144.37, 143.51, 133.66, 131.90, 131.74, 130.12, 127.79, 127.20; Melting point: 205–207 °C; Elemental analysis calculated for $C_{12}H_9ClN_4O$ (MW 260.68): 55.29% C, 3.48% H, 21.49% N, measured: 54.86% C, 3.27% H, 21.42% N; CAS Registry Number: 935886-78-5[21-23]

(*E*)-*N*'-(3-chlorobenzylidene)pyrazine-2-carbohydrazide (2)

White solid, yield 85%; ^{1}H NMR (600 MHz, DMSO-d_6) δ 12.38 (s, 1H), 9.23 (d, *J* = 1.5 Hz, 1H), 8.90 (d, *J* = 2.5 Hz, 1H), 8.76 (t, *J* = 2.1 Hz, 1H), 8.60 (s, 1H), 7.73 (s, 1H), 7.64 (dt, *J* = 6.5, 1.9 Hz, 1H), 7.53–7.42 (m, 2H); ^{13}C NMR (151 MHz, DMSO-d_6) δ 160.25, 148.69, 148.46, 145.06, 144.73, 143.87, 136.92, 134.23, 131.37, 130.53, 126.91, 126.58; Melting point: 197–198 °C; Elemental analysis calculated for $C_{12}H_9ClN_4O$ (MW 260.68): 55.29% C, 3.48% H, 21.49% N, measured: 55.03% C, 3.17% H, 21.13% N; CAS Registry Number: 1206198-44-8[22-23]

(*E*)-*N*'-(4-chlorobenzylidene)pyrazine-2-carbohydrazide (3)

White solid, yield 90%; ^{1}H NMR (500 MHz, DMSO-d_6) δ 12.35 (s, 1H), 9.26 (d, *J* = 1.4 Hz, 1H), 8.93 (d, *J* = 2.5 Hz, 1H), 8.81–8.77 (m, 1H), 8.64 (s, 1H), 7.80–7.72 (m, 2H), 7.61–7.50 (m, 2H); ^{13}C NMR (151 MHz, DMSO-d_6) δ 160.14, 149.27, 148.24, 145.33, 144.61, 143.76, 135.42, 133.75, 130.46, 129.46; Melting point: 251–253 °C; Elementary analysis calculated for $C_{12}H_9ClN_4O$ (MW 260.68): 55.29% C, 3.48% H, 21.49% N, measured: 54.94% C, 3.24% H, 21.19% N; CAS Registry Number: 926303-83-5[22-24]

(*E*)-*N*'-(2-bromobenzylidene)pyrazine-2-carbohydrazide (4)

White solid, yield 82%; ^{1}H NMR (500 MHz, DMSO-d_6) δ 12.65 (s, 1H), 9.27 (d, *J* = 1.5 Hz, 1H), 9.04 (s, 1H), 8.92 (d, *J* = 2.5 Hz, 1H), 8.79 (dd, *J* = 2.4, 1.5 Hz, 1H), 8.02 (dd, *J* = 7.9, 1.8 Hz, 1H), 7.69 (dd, *J* = 8.0, 1.2 Hz, 1H), 7.48 (m, *J* = 7.8, 7.2, 1.3, 0.7 Hz, 1H), 7.38 (m, *J* = 8.1, 7.3, 1.8 Hz, 1H); ^{13}C NMR (126 MHz, DMSO-d_6) δ 160.01, 148.37, 148.06, 144.76, 144.38, 143.51, 133.37, 133.26, 132.14, 128.28, 127.60, 123.98; Melting point: 233–

235 °C; Elementary analysis calculated for $C_{12}H_9BrN_4O$ (MW 305.14): 47.24% C, 2.97% H, 18.36% N, measured: 47.49% C, 2.81% H, 18.56% N; CAS Registry Number: 1206198-48-2[23]

(*E*)-*N*'-(3-bromobenzylidene)pyrazine-2-carbohydrazide (5)

White solid, yield 80%; ^{1}H NMR (500 MHz, DMSO-*d6*) δ 12.41 (s, 1H), 9.27 (d, *J* = 1.5 Hz, 1H), 8.93 (d, *J* = 2.5 Hz, 1H), 8.79 (dd, *J* = 2.5, 1.5 Hz, 1H), 8.61 (s, 1H), 7.91 (t, *J* = 1.8 Hz, 1H), 7.71 (dt, *J* = 7.8, 1.3 Hz, 1H), 7.64 (m, *J* = 8.0, 2.1, 1.0 Hz, 1H), 7.43 (t, *J* = 7.9 Hz, 1H); ^{13}C NMR (126 MHz, DMSO-*d6*) δ 159.87, 148.22, 148.09, 144.68, 144.36, 143.50, 136.77, 133.04, 131.25, 129.39, 126.62, 122.37; Melting point: 212–213 °C; Elemental analysis calculated for $C_{12}H_9BrN_4O$ (MW 305.14): 47.24% C, 2.97% H, 18.36% N, measured: 47.23% C, 2.73% H, 18.4% N; CAS Registry Number: 1206198-49-3[22-23, 25]

(*E*)-*N*'-(4-bromobenzylidene)pyrazine-2-carbohydrazide (6)

White solid, yield 82%; ^{1}H NMR (500 MHz, DMSO-*d6*) δ 12.36 (s, 1H), 9.26 (d, *J* = 1.5 Hz, 1H), 8.93 (d, *J* = 2.5 Hz, 1H), 8.81–8.76 (m, 1H), 8.62 (s, 1H), 7.68 (s, 4H); ^{13}C NMR (151 MHz, DMSO-*d6*) δ 160.14, 149.13, 148.44, 145.11, 144.70, 143.87, 133.97, 132.47, 129.66, 124.17; Melting point: 273–275 °C; Elemental analysis calculated for $C_{12}H_9BrN_4O$ (MW 305.14): 47.24% C, 2.97% H, 18.36% N, measured: 47.35% C, 2.75% H, 18.43% N; CAS Registry Number: 1206198-50-6[22-23, 25]

(*E*)-*N*'-(2-fluorobenzylidene)pyrazine-2-carbohydrazide (7)

White solid, yield 88%; ^{1}H NMR (500 MHz, DMSO-*d6*) δ 12.49 (s, 1H), 9.27 (d, *J* = 1.5 Hz, 1H), 8.96–8.88 (m, 2H), 8.79 (dd, *J* = 2.6, 1.5 Hz, 1H), 7.96 (td, *J* = 7.7, 1.9 Hz, 1H), 7.55–7.46 (m, 1H), 7.34–7.26 (m, 2H); ^{13}C NMR (151 MHz, DMSO-*d6*) δ 162.29, 160.43 (d, *J* = 60.8 Hz), 148.45, 145.06, 144.73, 143.87, 143.14 (d, *J* = 4.5 Hz), 132.85 (d, *J* = 8.6 Hz), 127.03, 125.51, 122.34 (d, *J* = 10.0 Hz), 116.59 (d, *J* = 20.3 Hz); Melting point: 216–218 °C; HPLC purity 99.81%; HRMS(ESI$^+$): [M$^+$H]$^+$ calculated for $C_{12}H_{10}FN_4O^+$ (m/z): 245.08332, found 245.08315; CAS Registry Number: 1206198-45-9[22-23]

(*E*)-*N*'-(3-fluorobenzylidene)pyrazine-2-carbohydrazide (8)

White solid, yield 86%; ^{1}H NMR (500 MHz, DMSO-*d6*) δ 12.41 (s, 1H), 9.27 (d, *J* = 1.5 Hz, 1H), 8.93 (d, *J* = 2.4 Hz, 1H), 8.82–8.77 (m, 1H), 8.65 (s, 1H), 7.58–7.49 (m, 3H), 7.34–7.26 (m, 1H); ^{13}C NMR (151 MHz, DMSO-*d6*) δ 163.85, 162.23, 149.23, 148.28, 145.31, 144.64, 143.79, 137.36, 131.42 (d, *J* = 7.8 Hz), 124.20, 117.52 (d, *J* = 21.4 Hz), 113.62 (d, *J* = 23.0 Hz); Melting point: 263–265 °C; HPLC purity 99.85%; HRMS(ESI$^+$): [M$^+$H]$^+$ calculated for $C_{12}H_{10}FN_4O^+$ (m/z): 245.08332, found 245.08316; CAS Registry Number: 1206198-46-0[23]

(*E*)-*N*'-(4-fluorobenzylidene)pyrazine-2-carbohydrazide (9)

White solid, yield 89%; ^{1}H NMR (500 MHz, DMSO-*d6*) δ 12.29 (s, 1H), 9.26 (d, *J* = 1.5 Hz, 1H), 8.92 (d, *J* = 2.5 Hz, 1H), 8.79 (dd, *J* = 2.5, 1.5 Hz, 1H), 8.64 (s, 1H), 7.84–7.75 (m, 2H), 7.35–7.27 (m, 2H); ^{13}C NMR (126 MHz,

DMSO-d_6) δ 164.44, 161.08 (d, *J* = 347.5 Hz), 148.86, 148.02, 144.80, 144.30, 143.49, 130.94 (d, *J* = 3.0 Hz), 129.65 (d, *J* = 8.6 Hz), 116.16 (d, *J* = 21.9 Hz).; Melting point: 244 -246 °C; HPLC purity 99.67%; HRMS(ESI$^+$): $[M^+H]^+$ calculated for $C_{12}H_{10}FN_4O^+$ (m/z): 245.08332, found 245.08305; CAS Registry Number: 1206198-47-1[22-23, 26]

(*E*)-*N*'-(2,3,6-trichlorobenzylidene)pyrazine-2-carbohydrazide (10)

White solid, yield 80%; ^{1}H NMR (500 MHz, DMSO-d_6) δ 12.72 (s, 1H), 9.27 (d, *J* = 1.5 Hz, 1H), 8.94 (d, *J* = 2.5 Hz, 1H), 8.85–8.78 (m, 2H), 7.74 (d, *J* = 8.7 Hz, 1H), 7.60 (d, *J* = 8.7 Hz, 1H); ^{13}C NMR (126 MHz, DMSO-d_6) δ 160.11, 148.18, 145.25, 144.54, 144.50, 143.49, 132.98, 132.61, 132.31, 131.68, 131.63, 129.98; Melting point: 214–216 °C; Elemental analysis calculated for $C_{12}H_7Cl_3N_4O$ (MW 329.57): 43.73% C, 2.14% H, 17.00% N, measured: 43.28% C, 1.89% H, 16.95% N

(*E*)-*N*'-(2-nitrobenzylidene)pyrazine-2-carbohydrazide (11)

Yellow solid, yield 91%; ^{1}H NMR (600 MHz, DMSO-d_6) δ 12.64 (s, 1H), 9.23 (d, *J* = 1.5 Hz, 1H), 9.03 (s, 1H), 8.89 (d, *J* = 2.5 Hz, 1H), 8.76 (t, *J* = 2.0 Hz, 1H), 8.09 (dd, *J* = 7.8, 1.4 Hz, 1H), 8.05 (dd, *J* = 8.2, 1.2 Hz, 1H), 7.80 (m, *J* = 7.7, 1.2 Hz, 1H), 7.69 – 7.63 (m, 1H).); ^{13}C NMR (151 MHz, DMSO-d_6) δ 160.56, 148.94, 148.50, 145.58, 145.04, 144.79, 143.90, 134.30, 131.50, 129.09, 128.68, 125.18; Melting point: 284 -286 °C; Elemental analysis calculated for $C_{12}H_9N_5O_3$ (MW 271.24): 53.14% C, 3.34% H, 25.82% N, measured: 52.71% C, 3.13% H, 25.83% N; CAS Registry Number: 1206198-61-9[23, 27]

(*E*)-*N*'-(3-nitrobenzylidene)pyrazine-2-carbohydrazide (12)

Yellow solid, yield 88%; ^{1}H NMR (500 MHz, DMSO-d_6) δ 12.53 (s, 1H), 9.27 (d, *J* = 1.5 Hz, 1H), 8.93 (d, *J* = 2.5 Hz, 1H), 8.80 (dd, *J* = 2.5, 1.5 Hz, 1H), 8.75 (s, 1H), 8.51 (t, *J* = 2.0 Hz, 1H), 8.27 (m, *J* = 8.2, 2.4, 1.0 Hz, 1H), 8.13 (dt, *J* = 7.8, 1.3 Hz, 1H), 7.75 (t, *J* = 8.0 Hz, 1H); ^{13}C NMR (126 MHz, DMSO-d_6) δ 160.00, 148.40, 148.16, 147.58, 144.58, 144.41, 143.52, 136.14, 133.68, 130.72, 124.70, 121.24; Melting point: 284–285 °C; Elemental analysis calculated for $C_{12}H_9N_5O_3$ (MW 271.24): 53.14% C, 3.34% H, 25.82% N, measured: 52.87% C, 3.18% H, 25.56% N; CAS Registry Number: 1206198-62-0[23]

(*E*)-*N*'-(4-nitrobenzylidene)pyrazine-2-carbohydrazide (13)

Yellow solid, yield 82%; ^{1}H NMR (500 MHz, DMSO-d_6) δ 12.59 (s, 1H), 9.28 (d, *J* = 1.5 Hz, 1H), 8.95 (d, *J* = 2.5 Hz, 1H), 8.81 (dd, *J* = 2.4, 1.5 Hz, 1H), 8.76 (s, 1H), 8.35–8.28 (m, 2H), 8.04–7.96 (m, 2H); ^{13}C NMR (151 MHz, DMSO-d_6) δ 160.47, 148.60, 147.90, 144.92, 144.81, 143.92, 140.94, 131.56, 128.76, 124.69; Melting point: 283–284 °C; Elemental analysis calculated for $C_{12}H_9N_5O_3$ (MW 271.24): 53.14% C, 3.34% H, 25.82% N, measured: 52.82% C, 3.15% H, 25.77% N; CAS Registry Number: 1206198-63-1[23, 26]

(*E*)-*N*'-(2-methoxybenzylidene)pyrazine-2-carbohydrazide (14)

White powder, yield 94%; ^{1}H NMR (500 MHz, DMSO-d_6) δ 12.32 (s, 1H), 9.25 (d, *J* = 1.5 Hz, 1H), 8.98 (d, *J* = 0.7 Hz, 1H), 8.90 (d, *J* = 2.5 Hz, 1H), 8.80–8.75 (m, 1H), 7.88 (dd, *J* = 7.7, 1.8 Hz, 1H), 7.43 (m, *J* = 7.4, 1.8, 0.9 Hz, 1H), 7.11 (dd, *J* = 8.4, 1.0 Hz, 1H), 7.08–6.99 (m, 1H), 3.86 (s, 3H); ^{13}C NMR (126 MHz, DMSO-d_6) δ 159.72, 158.17, 147.87, 145.63, 145.02, 144.26, 143.49, 132.03, 125.90, 122.46, 120.92, 112.10, 55.94; Melting point: 209–210 °C; Elemental analysis calculated for: $C_{13}H_{12}N_4O_2$ (MW 256.27): 60.93% C, 4.72% H, 21.86% N, measured: 60.58% C, 4.6% H, 21.79% N; CAS Registry Number: 1206198-51-7[23, 28]

(*E*)-*N*'-(4-hydroxy-3-methoxybenzylidene)pyrazine-2-carbohydrazide (15)

Pale yellow solid, yield 84%; ^{1}H NMR (500 MHz, DMSO-d_6) δ 12.08 (s, 1H), 9.58 (s, 1H), 9.25 (d, *J* = 1.4 Hz, 1H), 8.91 (d, *J* = 2.5 Hz, 1H), 8.77 (dd, *J* = 2.5, 1.5 Hz, 1H), 8.51 (s, 1H), 7.31 (d, *J* = 1.9 Hz, 1H), 7.08 (dd, *J* = 8.3, 1.9 Hz, 1H), 6.85 (d, *J* = 8.1 Hz, 1H), 3.84 (s, 3H); ^{13}C NMR (126 MHz, DMSO-d_6) δ 159.34, 150.52, 149.47, 148.24, 147.85, 145.02, 144.19, 143.44, 125.70, 122.65, 115.66, 109.29, 55.75; Melting point: 250–251 °C; Elemental analysis calculated for $C_{13}H_{12}N_4O_3$ (MW 272.26): 57.35% C, 4.44% H, 20.58% N, measured: 57.03% C, 4.15% H, 20.23% N. CAS Registry Number: 304908-43-8[22, 29-30]

(*E*)-*N*'-(3-methoxybenzylidene)pyrazine-2-carbohydrazide (16)

White powder, yield 90%; ^{1}H NMR (500 MHz, DMSO-d_6) δ 12.29 (s, 1H), 9.26 (d, *J* = 1.5 Hz, 1H), 8.92 (d, *J* = 2.5 Hz, 1H), 8.79 (dd, *J* = 2.5, 1.5 Hz, 1H), 8.62 (s, 1H), 7.43–7.35 (m, 1H), 7.31–7.25 (m, 2H), 7.03 (m, *J* = 8.2, 2.5, 1.2 Hz, 1H), 3.81 (s, 3H); ^{13}C NMR (126 MHz, DMSO-d_6) δ 159.74, 159.72, 149.91, 148.01, 144.82, 144.31, 143.49, 135.74, 130.19, 120.44, 116.70, 111.50, 55.38; Melting point: 187–189 °C; Elemental analysis calculated for $C_{13}H_{12}N_4O_2$ (MW 256.27): 60.93% C, 4.72% H, 21.86% N, measured: 60.56% C, 4.57% H, 21.45% N; CAS Registry Number: 1206198-52-8[23, 27]

(*E*)-*N*'-(4-methoxybenzylidene)pyrazine-2-carbohydrazide (17)

White solid, yield 92%; ^{1}H NMR (500 MHz, DMSO-d_6) δ 12.14 (s, 1H, CONH), 9.25 (d, *J* = 1.5 Hz, 1H, ArH), 8.91 (d, *J* = 2.5 Hz, 1H), 8.77 (dd, *J* = 2.5, 1.5 Hz, 1H), 8.57 (s, 1H, CH_2), 7.72–7.62 (m, 2H), 7.08–6.99 (m, 2H), 3.81 (s, 3H); ^{13}C NMR (126 MHz, DMSO-d_6) δ 161.25, 159.45, 149.88, 147.91, 144.95, 144.23, 143.46, 129.11, 126.86, 114.57, 55.51; Melting point: 225–226 °C; Elemental analysis: calculated for $C_{13}H_{12}N_4O_2$ (MW 256.27): 60.93% C, 4.72% H, 21.86% N, measured: 60.37% C, 4.49% H, 21.89% N; CAS Registry Number: 1206198-53-9[23, 27]

(*E*)-*N*'-(2-hydroxybenzylidene)pyrazine-2-carbohydrazide (18)

Light brown solid, yield 95%; IR (ATR-Ge, cm^{-1}): 3297 (phenolic -OH stretch), 3057 (amidic -NH stretch), 1668 (C=O), 1605 (C=N), 1529 (N-N), 1462, 1406, 1375 (aromatic C-C stretch); ^{1}H NMR (500 MHz, DMSO-d_6) δ 12.61 (s, 1H), 11.26 (s, 1H), 9.27 (d, *J* = 1.5 Hz, 1H), 8.93 (d, *J* = 2.7 Hz, 1H), 8.84 (s, 1H), 8.79 (m, *J* = 2.2, 1.5, 0.6 Hz,

1H), 7.51 (dd, *J* = 7.6, 1.7 Hz, 1H), 7.32 (m, *J* = 8.4, 7.3, 1.7 Hz, 1H), 6.98–6.87 (m, 2H); ^{13}C NMR (126 MHz, DMSO-d_6) δ 159.65, 157.81, 150.54, 148.13, 144.47, 144.37, 143.56, 131.87, 129.91, 119.58, 118.75, 116.67; Melting point: 207–209 °C; Elemental analysis calculated for $C_{12}H_{10}N_4O_2$ (MW 242.24): 59.50% C, 4.16% H, 23.13% N, measured: 59.5% C, 4.16% H, 23.13% N; HPLC purity 98.62%; HRMS(ESI$^+$): [M$^+$H]$^+$ calculated for $C_{12}H_{11}N_4O_2^+$ (m/z): 243.08765, found: 243.08745; CAS Registry Number: 1206198-56-2[23, 26]

(*E*)-*N*'-(3-hydroxybenzylidene)pyrazine-2-carbohydrazide (19)

White solid, yield 93%; ^{1}H NMR (500 MHz, DMSO-d_6) δ 12.22 (s, 1H), 9.64 (s, 1H), 9.26 (d, *J* = 1.5 Hz, 1H), 8.92 (d, *J* = 2.5 Hz, 1H), 8.80–8.76 (m, 1H), 8.56 (s, 1H), 7.26 (t, *J* = 7.8 Hz, 1H), 7.21 (t, *J* = 2.0 Hz, 1H), 7.09 (dt, *J* = 7.6, 1.3 Hz, 1H), 6.85 (m, *J* = 8.1, 2.5, 1.0 Hz, 1H); ^{13}C NMR (126 MHz, DMSO-d_6) δ 159.65, 157.88, 150.12, 147.99, 144.83, 144.29, 143.47, 135.59, 130.12, 119.17, 117.91, 112.96; Melting point: 259–261 °C; Elemental analysis calculated for $C_{12}H_{10}N_4O_2$ (MW 242.24): 59.50% C, 4.16% H, 23.13% N, measured: 59.01% C, 4.04% H, 23.02% N; CAS Registry Number: 1135685-44-7[23]

(*E*)-*N*'-(4-hydroxybenzylidene)pyrazine-2-carbohydrazide (20)

White solid, yield 96%; ^{1}H NMR (500 MHz, DMSO-d_6) δ 12.06 (s, 1H), 9.97 (s, 1H), 9.25 (d, *J* = 1.5 Hz, 1H), 8.90 (d, *J* = 2.4 Hz, 1H), 8.76 (dd, *J* = 2.5, 1.6 Hz, 1H), 8.53 (s, 1H), 7.63–7.53 (m, 2H), 6.89–6.81 (m, 2H); ^{13}C NMR (126 MHz, DMSO-d_6) δ 159.87, 159.34, 150.30, 147.86, 145.01, 144.21, 143.44, 129.29, 125.30, 115.95; Melting point: > 300 °C; Elemental analysis calculated for $C_{12}H_{10}N_4O_2$ (MW 242.24): 59.50% C, 4.16% H, 23.13% N, measured: 59.13% C, 3.9% H, 23.02% N; CAS Registry Number: 1206198-57-3[23, 26]

(*E*)-*N*'-(2,3-dihydroxybenzylidene)pyrazine-2-carbohydrazide (21)

Pale yellow solid, yield 93%; IR (ATR-Ge, cm^{-1}): 3324 (phenolic -OH stretch), 3293 (amidic -NH stretch), 1670 (C=O), 1614 (C=N), 1525 (N-N), 1461, 1403, 1358 (aromatic C-C stretch); ^{1}H NMR (600 MHz, DMSO-d_6) δ 12.59 (s, 1H), 11.11 (s, 1H), 9.24 (d, *J* = 1.4 Hz, 1H), 9.19 (s, 1H), 8.90 (d, *J* = 2.5 Hz, 1H), 8.76 (s, 2H), 6.89 (dd, *J* = 7.8, 1.6 Hz, 1H), 6.84 (dd, *J* = 7.8, 1.6 Hz, 1H), 6.72 (t, *J* = 7.8 Hz, 1H); ^{13}C NMR (126 MHz, DMSO-d_6) δ 159.62, 151.28, 148.14, 146.46, 145.82, 144.44, 144.38, 143.56, 120.47, 119.40, 118.81, 117.84; Melting point: 264–266 °C; Elemental analysis calculated for: $C_{12}H_{10}N_4O_3$ (MW 258.24): 55.81% C, 3.90% H, 21.70% N, measured: 55.4% C, 3.63% H, 21.61% N; HPLC purity 99.70%; HRMS(ESI$^+$): [M$^+$H]$^+$ calculated for $C_{12}H_{11}N_4O_3^+$ (m/z): 259.08257, found: 259.08246; CAS Registry Number: 1802157-73-8[30]

(*E*)-*N*'-(2,4-dihydroxybenzylidene)pyrazine-2-carbohydrazide (22)

White solid, yield 94%; IR (ATR-Ge, cm^{-1}): 3126 (phenolic -OH stretch), 3016 (amidic -NH stretch), 1663 (C=O), 1632 (C=N), 1509 (N-N), 1463, 1406, 1365 (aromatic C-C stretch); ^{1}H NMR (500 MHz, DMSO-d_6) δ 12.42 (s, 1H), 11.46 (s, 1H), 10.03–9.99 (m, 1H), 9.25 (d, *J* = 1.4 Hz, 1H), 8.91 (d, *J* = 2.5 Hz, 1H), 8.80–8.72 (m, 1H), 8.69 (s, 1H), 7.27 (d, *J* = 8.4 Hz, 1H), 6.37 (dd, *J* = 8.5, 2.3 Hz, 1H), 6.32 (d, *J* = 2.3 Hz, 1H); ^{13}C NMR (126 MHz, DMSO-

d$_6$) δ 161.20, 159.90, 159.24, 151.39, 148.01, 144.62, 144.28, 143.53, 131.84, 110.63, 108.03, 102.89; Melting point: 274–276 °C; Elemental analysis: calculated for $C_{12}H_{10}N_4O_3$ (MW 258.24): 55.81% C, 3.90% H, 21.70% N, measured: 55.41% C, 3.63% H, 21.43% N; HPLC purity 98.27%; HRMS(ESI$^+$): [M$^+$H]$^+$ calcd. for $C_{12}H_{11}N_4O_3^+$ (m/z): 259.08257, found: 259.08237; CAS Registry Number: 1323436-65-2[31]

(*E*)-*N*'-(4-hydroxy-3-nitrobenzylidene)pyrazine-2-carbohydrazide (23)

Yellow solid, yield 93%; ^{1}H NMR (500 MHz, DMSO-*d*$_6$) δ 12.31 (s, 1H), 11.48 (s, 1H), 9.25 (d, *J* = 1.5 Hz, 1H), 8.92 (d, *J* = 2.5 Hz, 1H), 8.78 (dd, *J* = 2.5, 1.5 Hz, 1H), 8.59 (s, 1H), 8.17 (d, *J* = 2.1 Hz, 1H), 7.92 (dd, *J* = 8.7, 2.1 Hz, 1H), 7.22 (d, *J* = 8.7 Hz, 1H); ^{13}C NMR (126 MHz, DMSO-*d*$_6$) δ 159.66, 153.74, 148.01, 147.94, 144.77, 144.30, 143.47, 137.24, 133.24, 125.79, 124.37, 119.96; Melting point: 254–256 °C; Elemental analysis calculated for $C_{12}H_9N_5O_4$ (MW 287.27): 50.18% C, 3.16% H, 24.38% N, measured: 49.88% C, 2.99% H, 24.35% N

(*E*)-*N*'-(pyridin-2-ylmethylene)pyrazine-2-carbohydrazide (24)

White solid, yield 87%; ^{1}H NMR (500 MHz, DMSO-*d*$_6$) δ 12.59 (s, 1H), 9.28 (d, *J* = 1.6 Hz, 1H), 8.94 (d, *J* = 2.5 Hz, 1H), 8.80 (dd, *J* = 2.5, 1.5 Hz, 1H), 8.68 (s, 1H), 8.62 (dt, *J* = 4.8, 1.5 Hz, 1H), 8.00 (dt, *J* = 7.9, 1.2 Hz, 1H), 7.89 (m, *J* = 7.7, 1.8 Hz, 1H), 7.43 (m, *J* = 7.5, 4.8, 1.2 Hz, 1H); ^{13}C NMR (151 MHz, DMSO-*d*$_6$) δ 160.42, 153.71, 150.51, 150.10, 148.52, 145.01, 144.82, 143.88, 137.46, 125.15, 120.65; Melting point: 207–209 °C; Elemental analysis calculated for $C_{11}H_9N_5O$ (MW 227.23): 58.14% C, 3.99% H, 30.82% N, measured: 57.71% C, 3.84% H, 30.69% N; CAS Registry number: 1239766-81-4[22, 26]

(*E*)-*N*'-(pyridin-3-ylmethylene)pyrazine-2-carbohydrazide (25)

White solid, yield 86%; ^{1}H NMR (500 MHz, DMSO-*d*$_6$) δ 12.46 (s, 1H), 9.27 (d, *J* = 1.5 Hz, 1H), 8.94 (d, *J* = 2.5 Hz, 1H), 8.85 (d, *J* = 2.2 Hz, 1H), 8.80 (dd, *J* = 2.5, 1.5 Hz, 1H), 8.70 (s, 1H), 8.63 (dd, *J* = 4.8, 1.7 Hz, 1H), 8.15 (m, *J* = 7.9, 1.9 Hz, 1H), 7.51 (dd, *J* = 7.9, 4.7 Hz, 1H); ^{13}C NMR (151 MHz, DMSO-*d*$_6$) δ 161.53, 152.67, 151.44, 149.30, 148.30, 145.22, 144.66, 143.81, 134.22, 130.70, 124.46; Melting point: 278–280 °C; Elemental analysis calculated for $C_{11}H_9N_5O$ (MW 227.23): 58.14% C, 3.99% H, 30.82% N, measured: 57.83% C, 3.81% H, 30.49% N

(*E*)-*N*'-(pyridin-4-ylmethylene)pyrazine-2-carbohydrazide (26)

White solid, yield 86%; ^{1}H NMR (500 MHz, DMSO-*d*$_6$) δ 12.57 (s, 1H), 9.28 (d, *J* = 1.4 Hz, 1H), 8.94 (d, *J* = 2.5 Hz, 1H), 8.80 (dd, *J* = 2.5, 1.5 Hz, 1H), 8.68–8.65 (m, 2H), 8.64 (s, 1H), 7.68–7.64 (m, 2H); ^{13}C NMR (151 MHz, DMSO-*d*$_6$) δ 160.46, 150.90, 148.59, 147.94, 144.91, 144.82, 143.91, 141.82, 121.65; Melting point: 247–249 °C; Elemental analysis calculated for $C_{11}H_9N_5O$ (MW 227.23): 58.14% C, 3.99% H, 30.82% N, measured 57.99% C, 3.87% H, 30.87% N; CAS Registry Number: 1239766-80-3[26]

(*E*)-*N*'-(furan-2-ylmethylene)pyrazine-2-carbohydrazide (27)

Beige solid, yield 82%; ^{1}H NMR (500 MHz, DMSO-d_6) δ 12.30 (s, 1H), 9.25 (d, *J* = 1.5 Hz, 1H), 8.92 (d, *J* = 2.5 Hz, 1H), 8.78 (dd, *J* = 2.5, 1.5 Hz, 1H), 8.53 (s, 1H), 7.87 (d, *J* = 1.7 Hz, 1H), 6.95 (d, *J* = 3.4 Hz, 1H), 6.65 (dd, *J* = 3.5, 1.8 Hz, 1H); ^{13}C NMR (126 MHz, DMSO-d_6) δ 159.61, 149.52, 148.01, 145.66, 144.72, 144.31, 143.44, 139.52, 114.21, 112.49; Melting point: 200–201 °C; Elemental analysis calculated for $C_{10}H_8N_4O_2$ (MW 216.20): 55.56% C, 3.73% H, 25.91% N, measured: 55.17% C, 3.32% H, 25.55% N; CAS Registry Number: 90920-35-7[30]

(*E*)-*N*'-(thiazol-2-ylmethylene)pyrazine-2-carbohydrazide (28)

White solid, yield 89%; ^{1}H NMR (500 MHz, DMSO-d_6) δ 12.73 (s, 1H), 9.28 (d, *J* = 1.5 Hz, 1H), 8.94 (d, *J* = 2.5 Hz, 1H), 8.87 (s, 1H), 8.82–8.76 (m, 1H), 8.01–7.94 (m, 1H), 7.90–7.85 (m, 1H); ^{13}C NMR (126 MHz, DMSO-d_6) δ 164.30, 159.95, 148.30, 144.50, 144.35, 144.35, 144.13, 143.54, 122.54; Melting point: 212–214 °C; Elemental analysis calculated for $C_9H_7N_5OS$ (MW 233.25): 46.34% C, 3.03% H, 30.03% N, 13.74% S, measured: 45.88% C, 2.66% H, 29.82% N, 13.95% S; CAS Registry Number: 1224623-79-3[32]

(*E*)-*N*'-(thiophen-2-ylmethylene)pyrazine-2-carbohydrazide (29)

White solid, yield 87%; ^{1}H NMR (500 MHz, DMSO-d_6) δ 12.28 (s, 1H), 9.25 (d, *J* = 1.5 Hz, 1H), 8.91 (d, *J* = 2.4 Hz, 1H), 8.83 (d, *J* = 0.7 Hz, 1H), 8.80–8.75 (m, 1H), 7.72–7.67 (m, 1H), 7.48–7.43 (m, 1H), 7.15 (dd, *J* = 5.0, 3.6 Hz, 1H); ^{13}C NMR (126 MHz, DMSO-d_6) δ 159.79, 148.26, 145.18, 145.08, 144.56, 143.75, 139.38, 131.82, 129.87, 128.42; Melting point: 205–207 °C; Elemental analysis calculated for $C_{10}H_8N_4OS$ (MW 232.26): 51.71% C, 3.47% H, 24.12% N, 13.80% S, measured: 51.27% C, 3.17% H, 23.91% N, 14.13% S; CAS Registry Number: 1297291-71-4[27]

(*E*)-*N*'-((3-methylthiophen-2-yl)methylene)pyrazine-2-carbohydrazide (30)

Grey solid, yield 91%; ^{1}H NMR (500 MHz, DMSO-d_6) δ 12.15 (s, 1H), 9.25 (d, *J* = 1.5 Hz, 1H), 8.97 (s, 1H), 8.91 (d, *J* = 2.5 Hz, 1H), 8.77 (dd, *J* = 2.4, 1.6 Hz, 1H), 7.59 (d, *J* = 5.0 Hz, 1H), 6.97 (d, *J* = 5.1 Hz, 1H), 2.32 (s, 3H); ^{13}C NMR (126 MHz, DMSO-d_6) δ 159.15, 147.95, 144.73, 144.26, 144.16, 143.42, 140.98, 132.49, 131.06, 128.62, 13.74; Melting point: 211–213 °C; Elemental analysis calculated for $C_{11}H_{10}N_4OS$ (MW 246.29): 53.64% C, 4.09% H, 22.75% N, 13.02% S, measured: 53.15% C, 3.82% H, 22.58% N, 13.33% S

(*E*)-*N*'-(2,2-diphenylethylidene)pyrazine-2-carbohydrazide (31)

White solid, yield 90%; ^{1}H NMR (500 MHz, DMSO-d_6) δ 12.05 (s, 1H), 9.22 (s, 1H), 8.89 (d, *J* = 2.5 Hz, 1H), 8.75 (s, 1H), 8.50 (d, *J* = 7.6 Hz, 1H), 7.36 (t, *J* = 7.5 Hz, 4H), 7.27 (dd, *J* = 13.0, 7.2 Hz, 6H), 5.04 (d, *J* = 7.6 Hz, 1H); ^{13}C NMR (151 MHz, DMSO-d_6) δ 159.92, 155.35, 148.35, 145.10, 144.59, 143.80, 141.76, 129.31, 128.74, 127.40, 53.59; Melting point: 215–217 °C; Elemental analysis calculated for $C_{19}H_{16}N_4O$ (MW 316.36): 72.13% C, 5.10% H, 17.71% N, measured: 72.03% C, 4.97% H, 17.92% N

N'-((1E,2E)-3-phenylallylidene)pyrazine-2-carbohydrazide (32)

Beige solid, yield: 93%; ^{1}H NMR (500 MHz, DMSO-d_6) δ 12.21 (s, 1H), 9.25 (d, J = 1.5 Hz, 1H), 8.91 (d, J = 2.5 Hz, 1H), 8.81–8.76 (m, 1H), 8.43 (d, J = 8.5 Hz, 1H), 7.71–7.61 (m, 2H), 7.43–7.37 (m, 2H), 7.37–7.30 (m, 1H), 7.10 (dd, J = 16.1, 8.5 Hz, 1H), 7.06 (s, 1H); ^{13}C NMR (126 MHz, DMSO-d_6) δ 159.57, 151.91, 147.94, 144.82, 144.30, 143.43, 139.97, 135.97, 129.16, 129.00, 127.37, 125.79; Melting point: 237–239 °C; Elemental analysis calculated for $C_{14}H_{12}N_4O$ (MW 252.28): 66.65% C, 4.79% H, 22.21% N, measured: 66.83% C, 4.85% H, 22.45% N; CAS Registry Number: 1164522-03-5[33]

(E)-N'-(cyclopropylmethylene)pyrazine-2-carbohydrazide (33)

Light brown solid, yield: 86%; ^{1}H NMR (600 MHz, DMSO-d_6) δ 11.72 (s, 1H), 9.17 (d, J = 1.7 Hz, 1H), 8.84 (d, J = 2.5 Hz, 1H), 8.69 (t, J = 2.0 Hz, 1H), 7.40 (d, J = 7.8 Hz, 1H), 1.66 (m, J = 12.7, 8.0, 4.6 Hz, 1H), 0.68–0.61 (m, 4H); ^{13}C NMR (151 MHz, DMSO-d_6) δ 159.23, 158.18, 148.13, 145.38, 144.52, 143.72, 14.22, 6.70; Melting point: 168–170 °C; Elemental analysis calculated for $C_9H_{10}N_4O$ (MW 190.21): 56.83% C, 5.30% H, 29.46% N, measured: 56.48% C, 5.03% H, 29.13% N

2.2 Representative NMR spectra (graphic)

2.2.1 ^{1}H NMR and ^{13}C NMR of compound **18**

^{1}H NMR

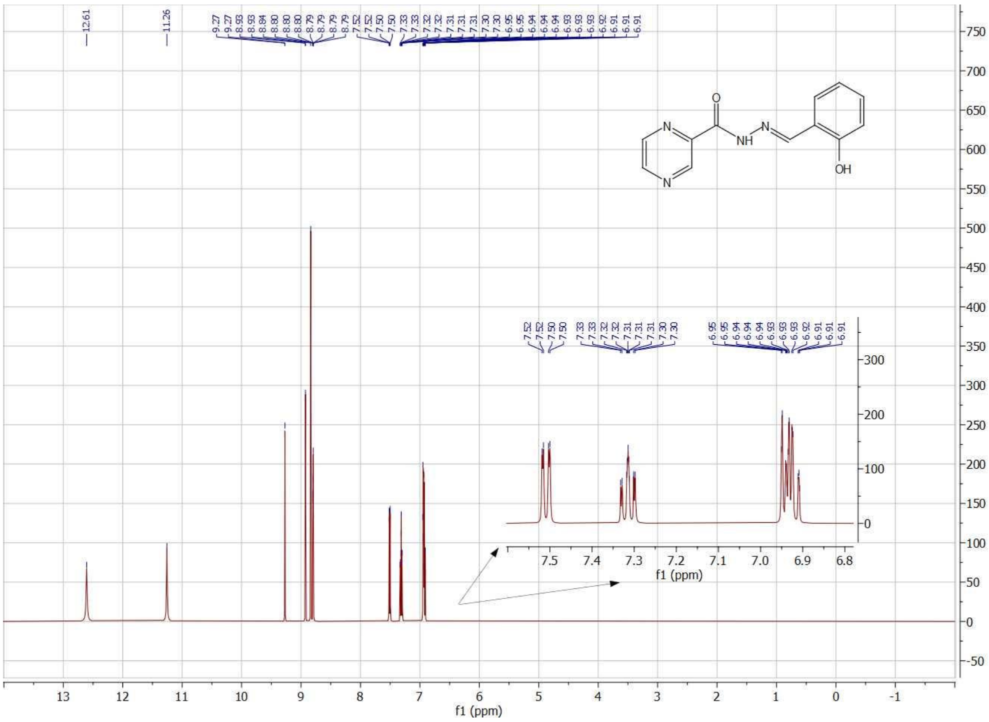

^{13}C NMR

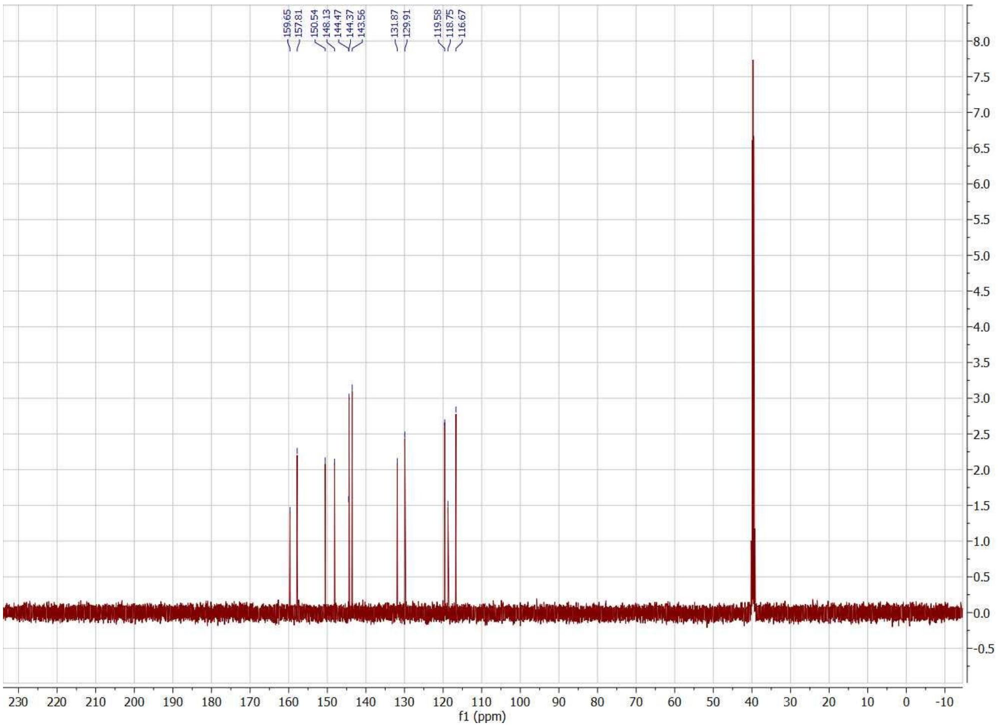

2.2.2 ^{1}H NMR and ^{13}C NMR of compound **21**

^{1}H NMR

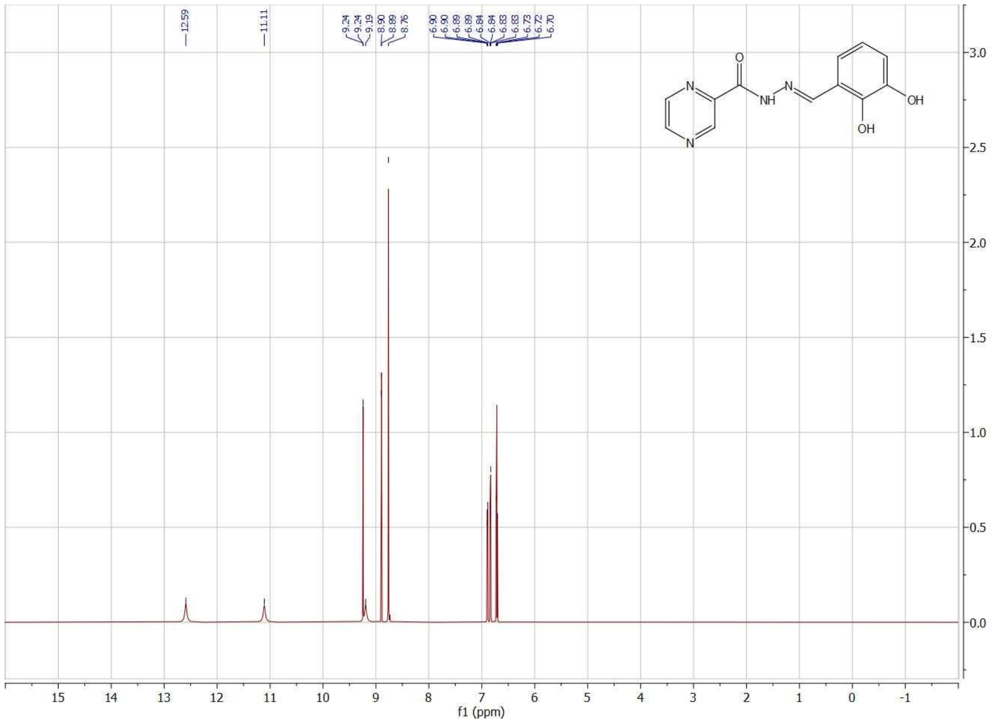

^{13}C NMR

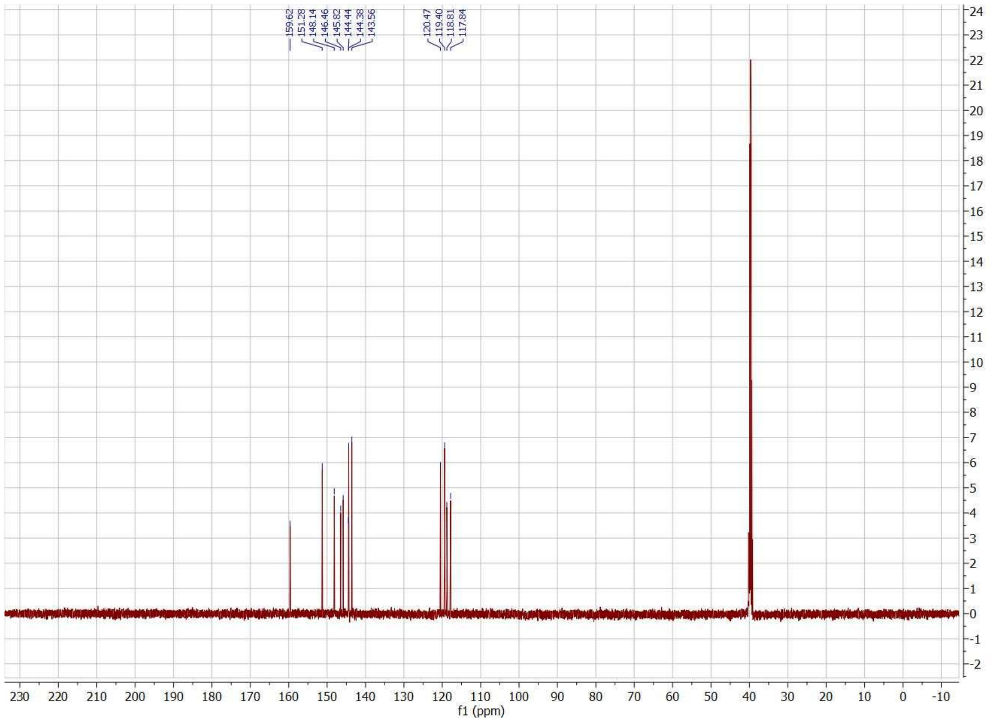

2.2.3 ^{1}H NMR and ^{13}C NMR of compound **22**

^{1}H NMR

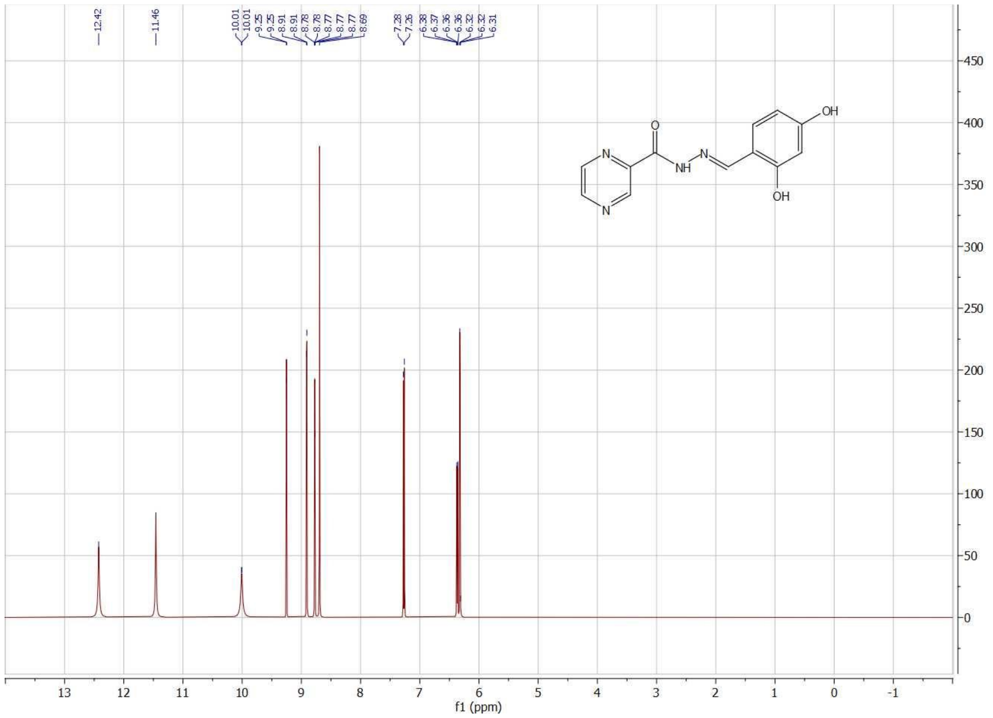

^{13}C NMR

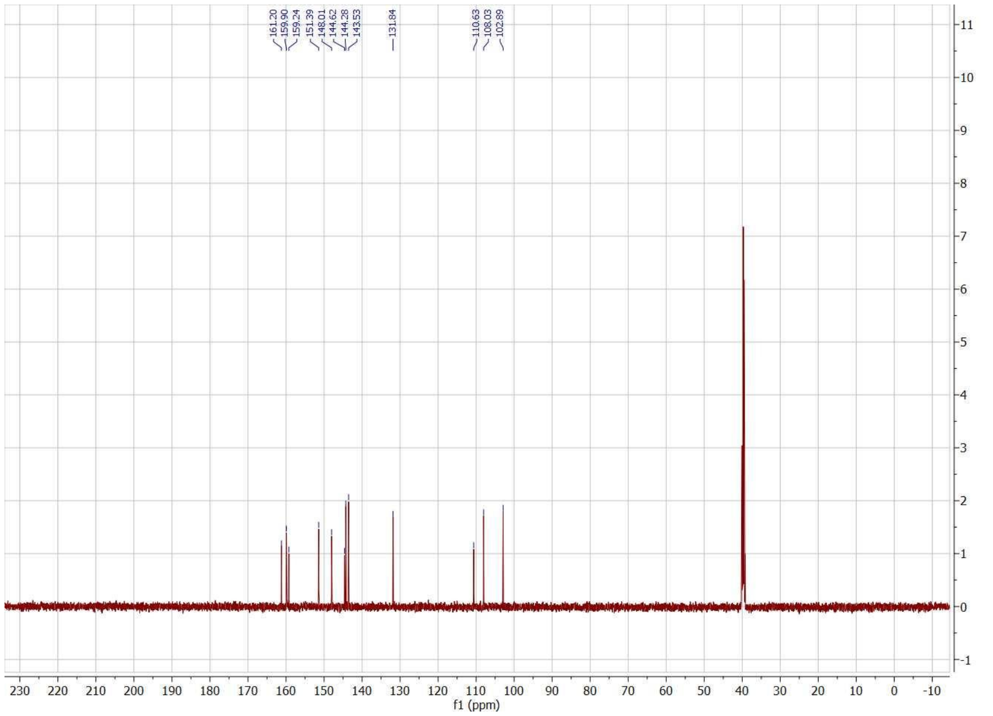

2.3 HPLC-HMRS analysis of fluorinated compounds and selected compounds with biological activity

Compound **7**

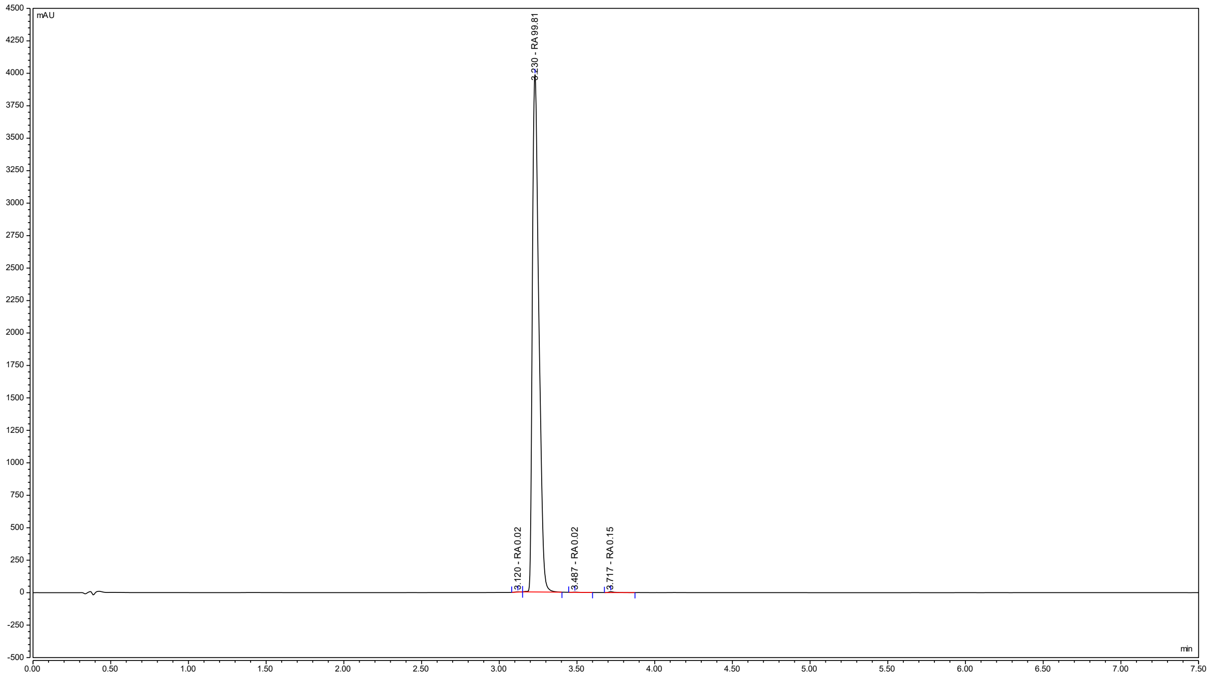

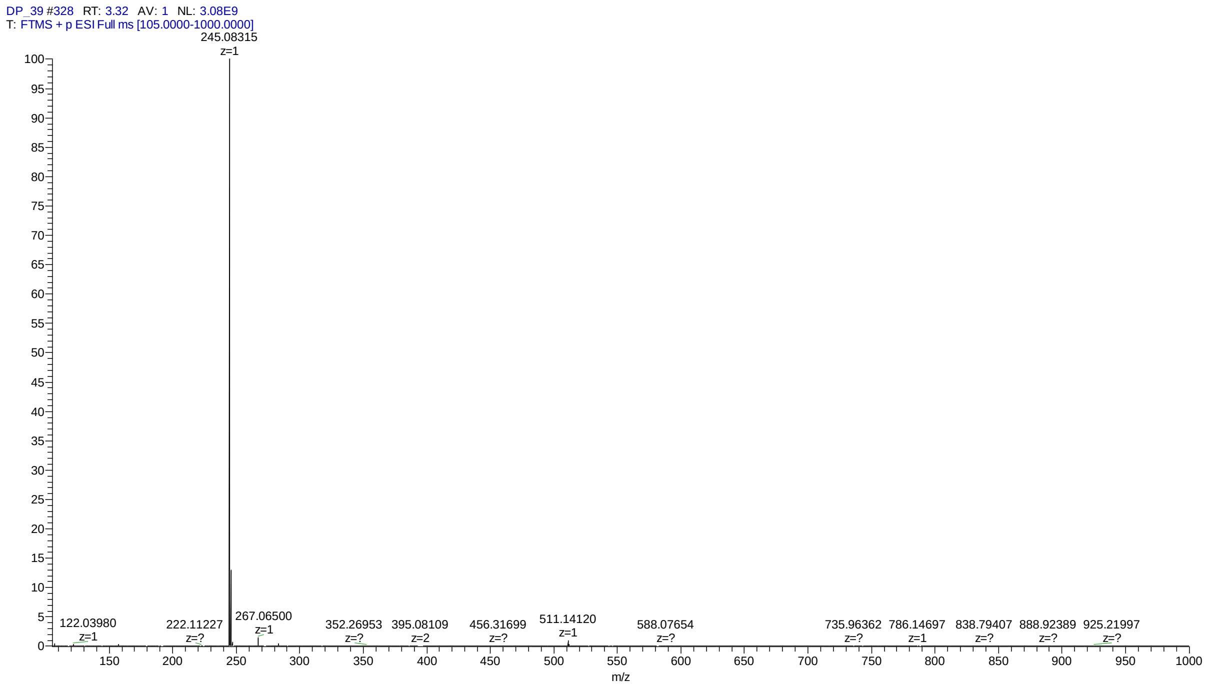

Compound **8**

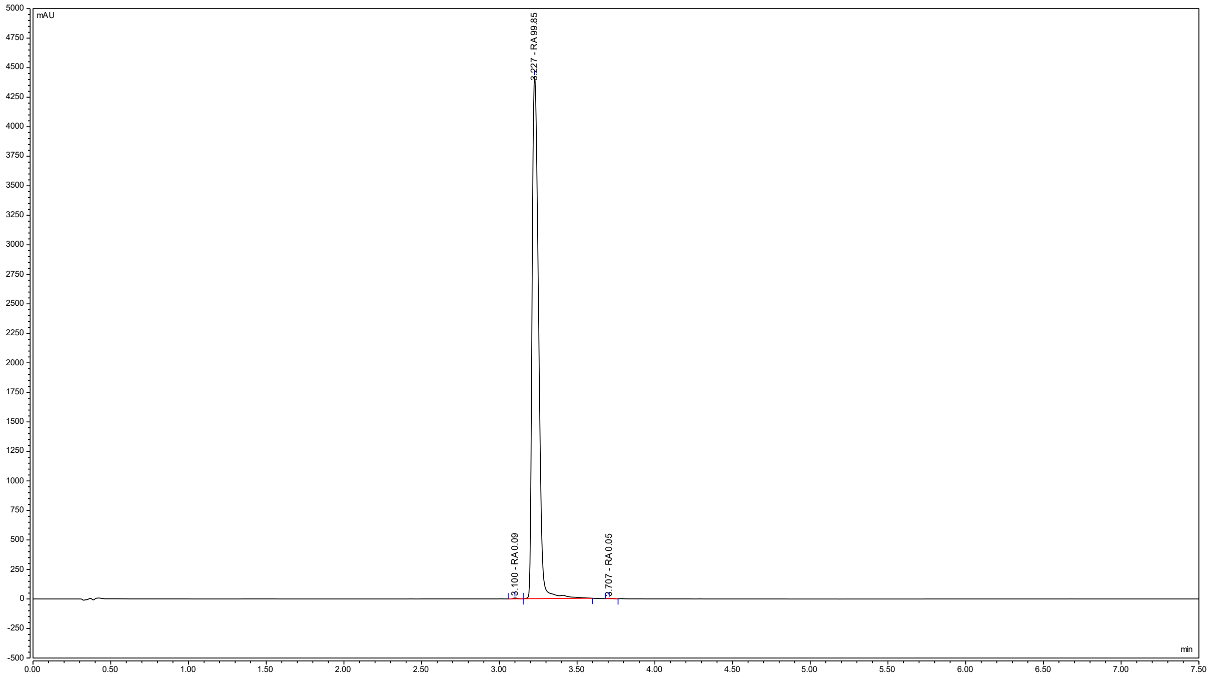
mAU
3.227 - RA 99.85
3.100 - RA 0.09
3.707 - RA 0.05
min

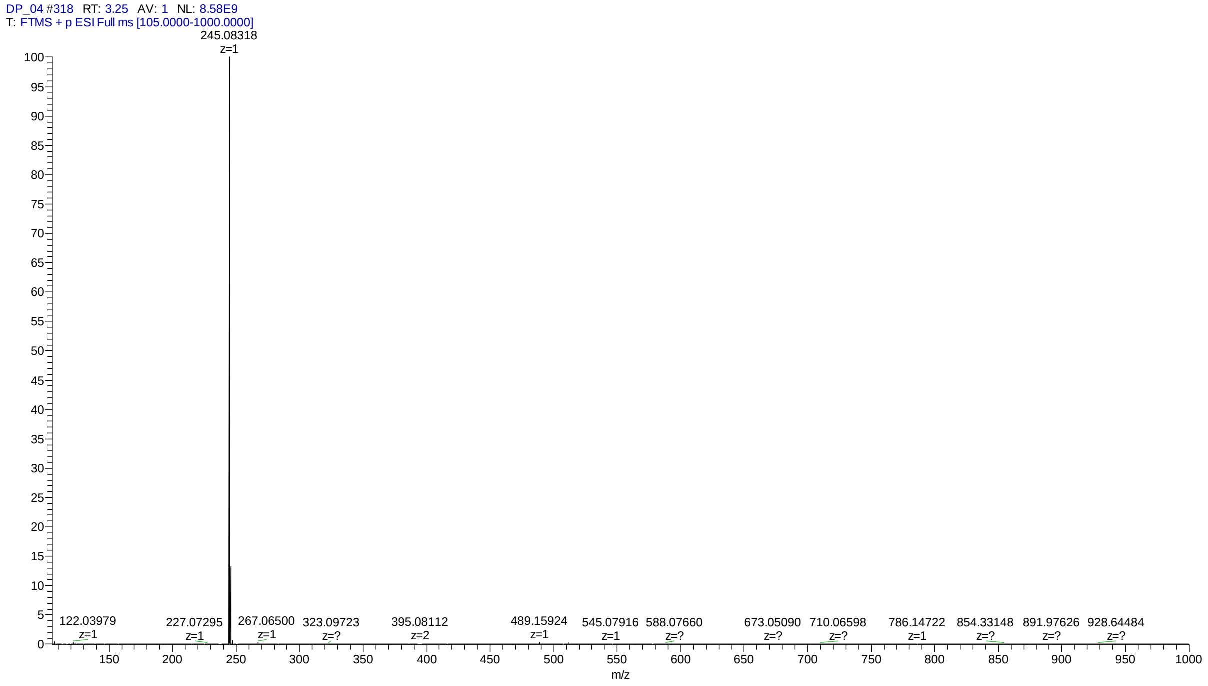
DP_04 #318 RT: 3.25 AV: 1 NL: 8.58E9
T: FTMS + p ESI Full ms [105.0000-1000.0000]
245.08318
z=1
122.03979 z=1
227.07295 z=1
267.06500 z=1
323.09723 z=?
395.08112 z=2
489.15924 z=1
545.07916 z=1
588.07660 z=?
673.05090 z=?
710.06598 z=?
786.14722 z=1
854.33148 z=?
891.97626 z=?
928.64484 z=?
m/z

Compound **9**

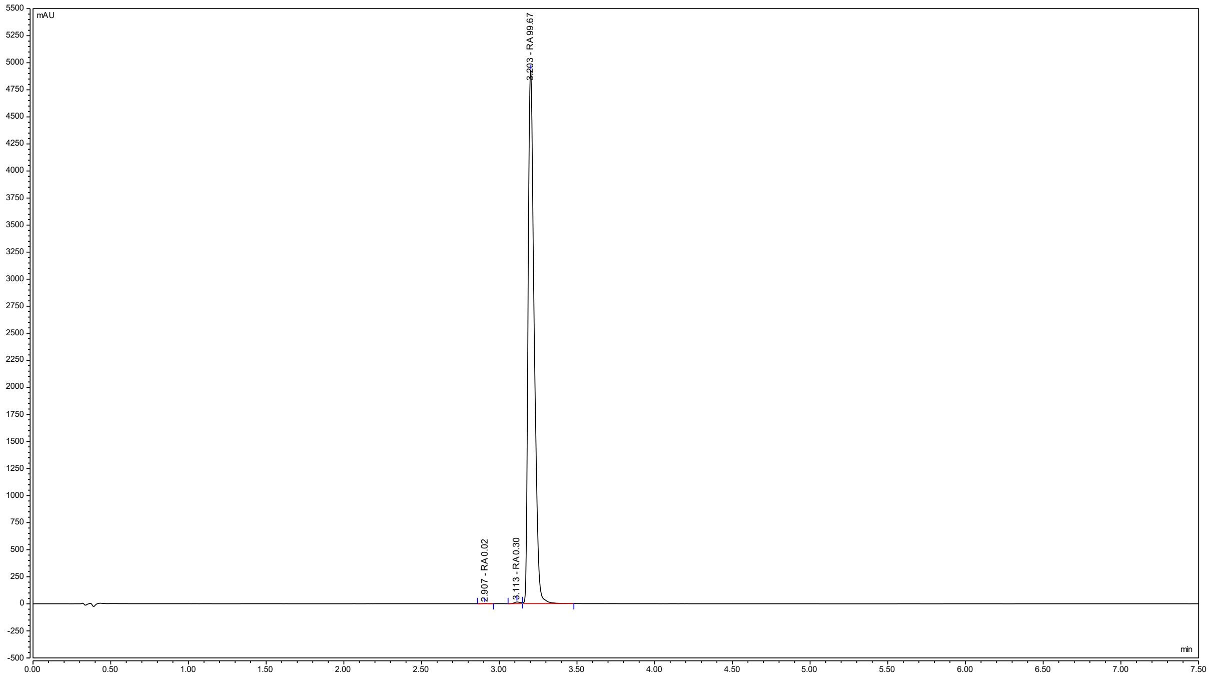
mAU
3.203 - RA 99.67
2.907 - RA 0.02
3.113 - RA 0.30
min

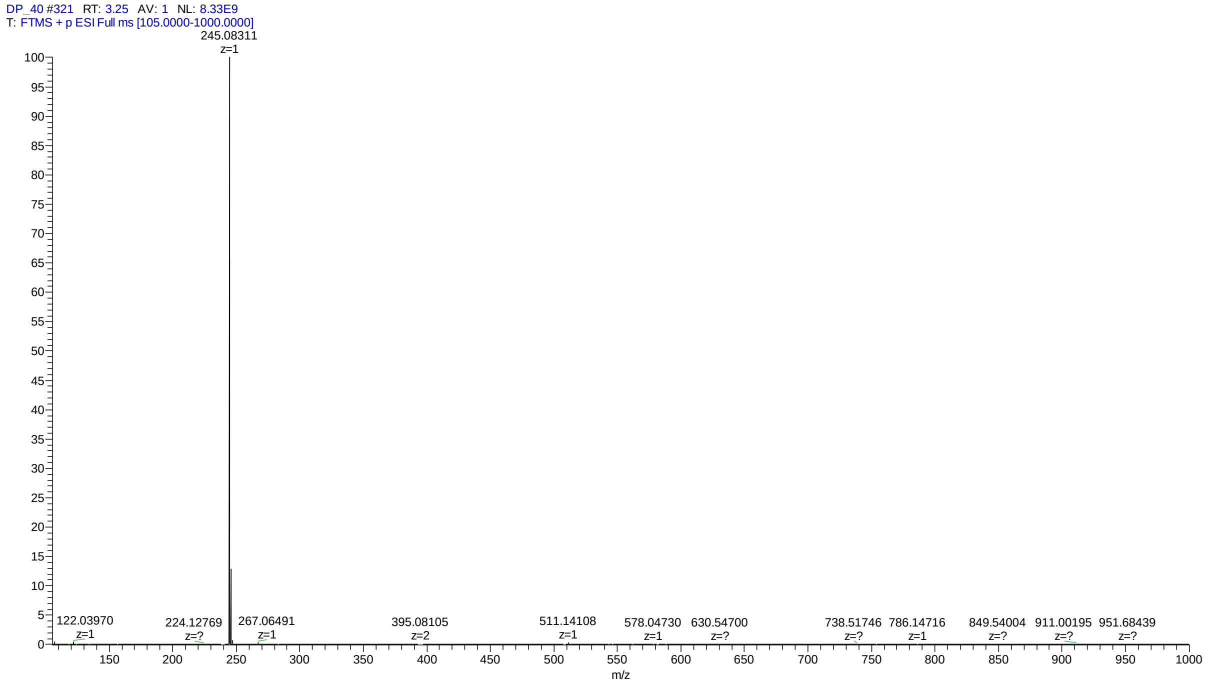
DP_40 #321 RT: 3.25 AV: 1 NL: 8.33E9
T: FTMS + p ESI Full ms [105.0000-1000.0000]
245.08311
z=1
122.03970
z=1
224.12769
z=?
267.06491
z=1
395.08105
z=2
511.14108
z=1
578.04730
z=1
630.54700
z=?
738.51746
z=?
786.14716
z=1
849.54004
z=?
911.00195
z=?
951.68439
z=?
m/z

Compound **18**

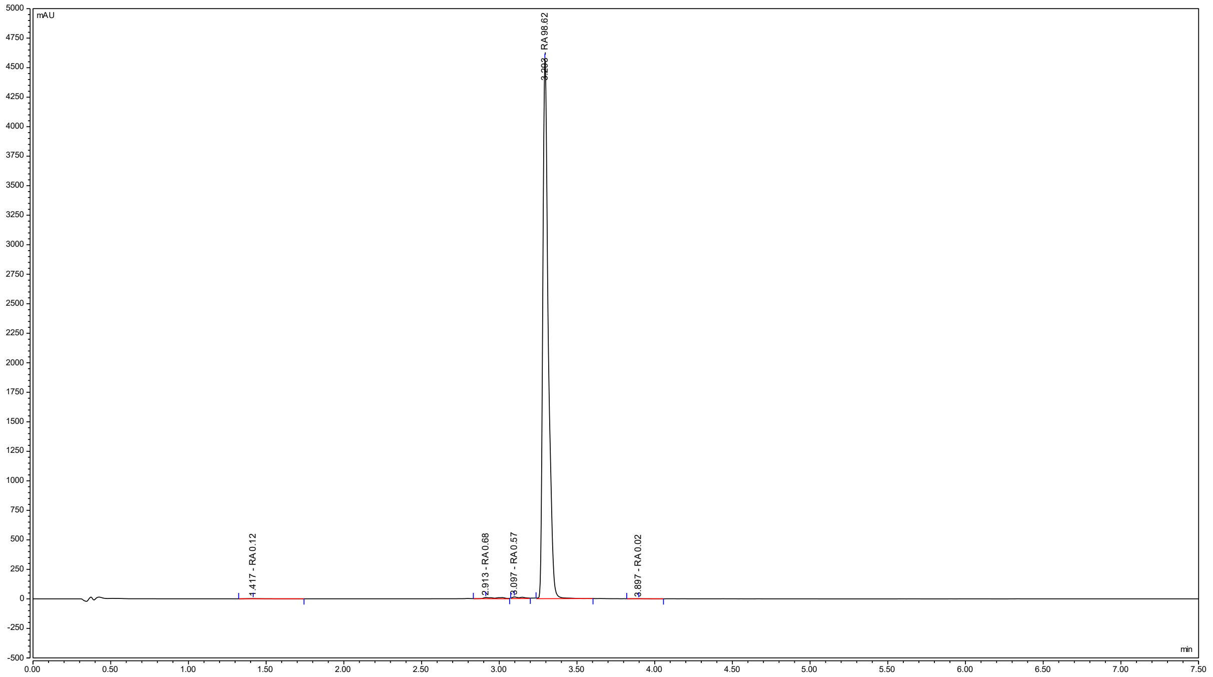

mAU
min
1.417 - RA 0.12
2.913 - RA 0.68
3.097 - RA 0.57
3.293 - RA 98.62
3.897 - RA 0.02

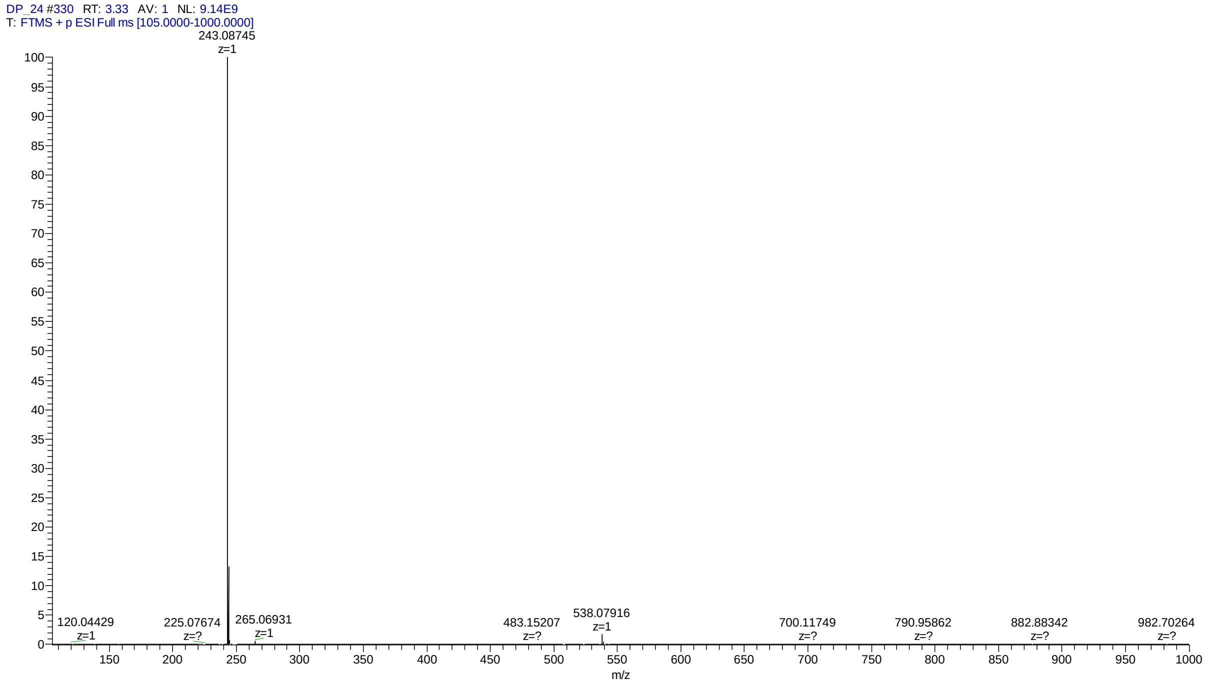

DP_24 #330 RT: 3.33 AV: 1 NL: 9.14E9
T: FTMS + p ESI Full ms [105.0000-1000.0000]
243.08745
z=1
120.04429
z=1
225.07674
z=?
265.06931
z=1
483.15207
z=?
538.07916
z=1
700.11749
z=?
790.95862
z=?
882.88342
z=?
982.70264
z=?
m/z

Compound **21**

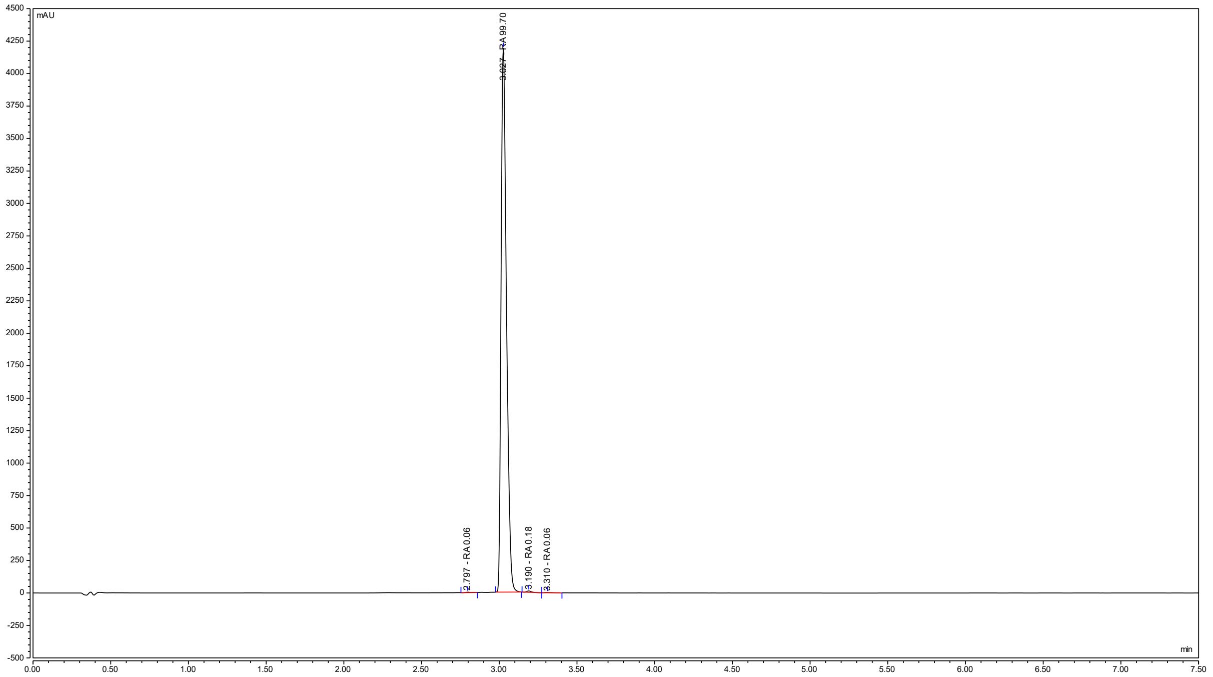
mAU
3.027 - RA99.70
2.797 - RA0.06
3.190 - RA0.18
3.310 - RA0.06
min

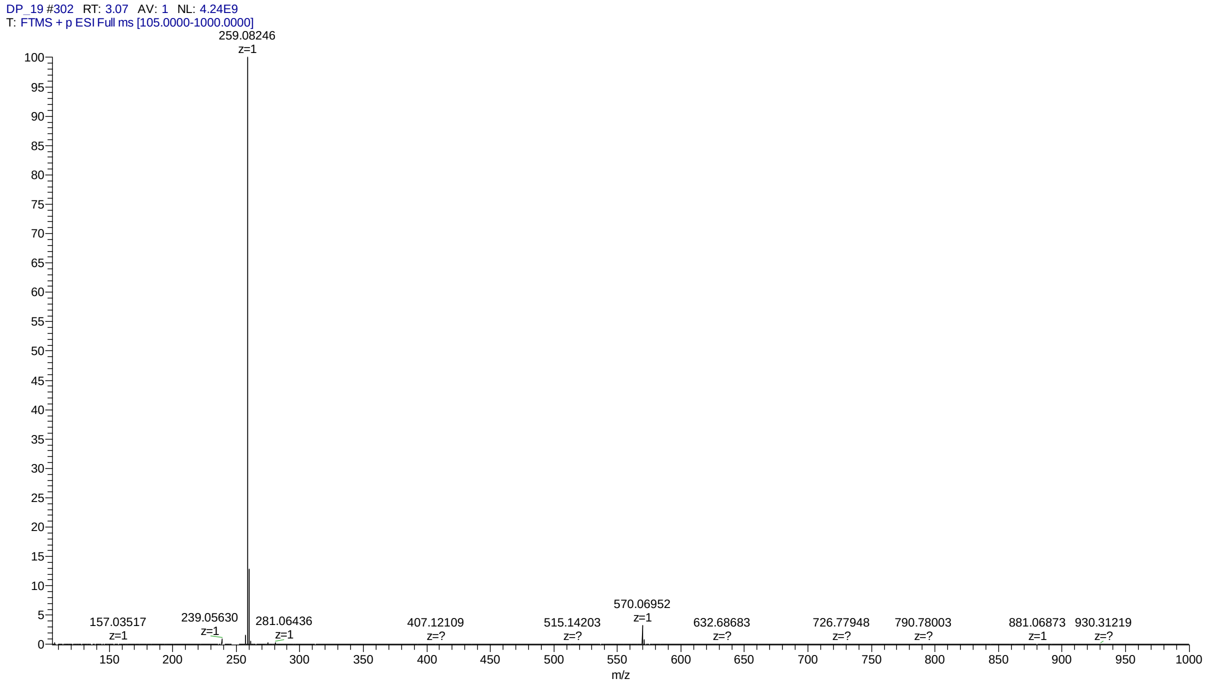
DP_19 #302 RT: 3.07 AV: 1 NL: 4.24E9
T: FTMS + p ESI Full ms [105.0000-1000.0000]
259.08246
z=1
157.03517
z=1
239.05630
z=1
281.06436
z=1
407.12109
z=?
515.14203
z=?
570.06952
z=1
632.68683
z=?
726.77948
z=?
790.78003
z=?
881.06873
z=1
930.31219
z=?
m/z

Compound **22**

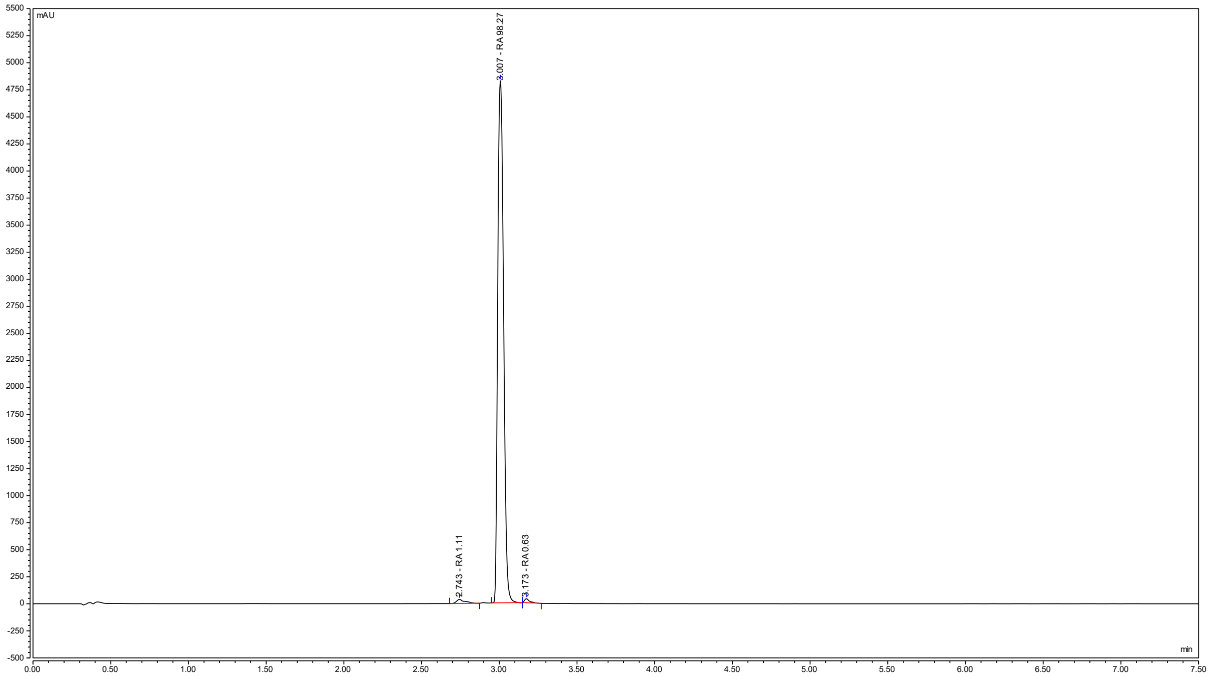
mAU
3.007 - RA 98.27
2.743 - RA 1.11
3.173 - RA 0.63
min

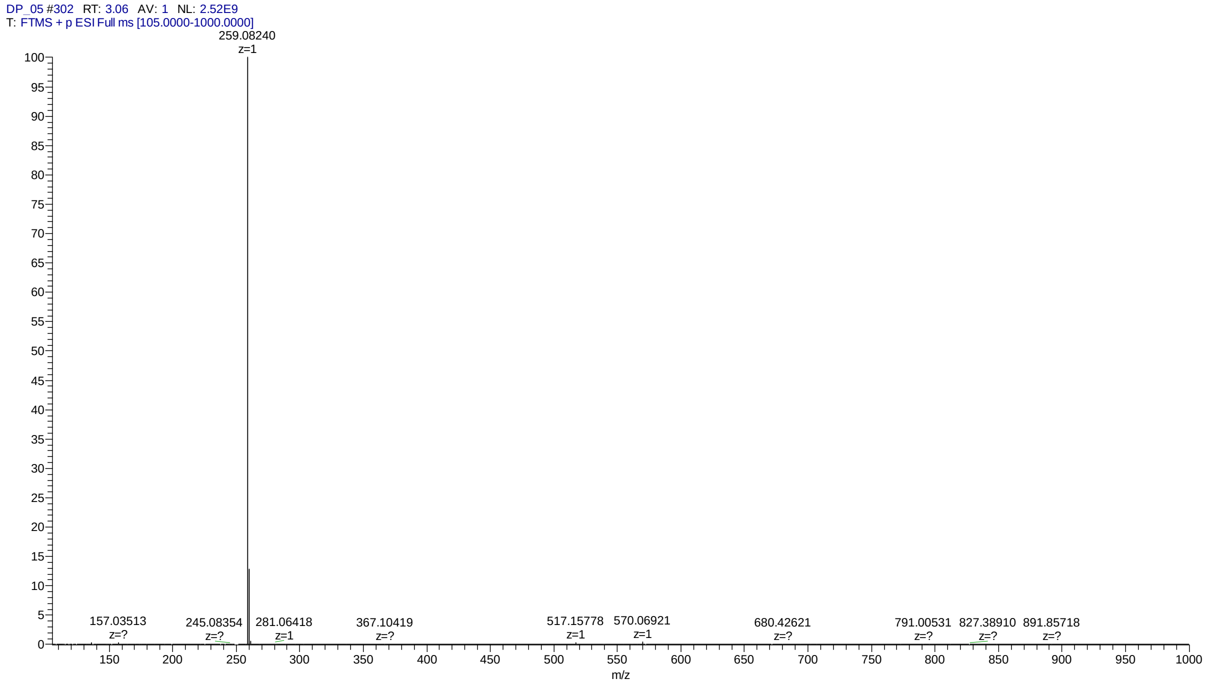
DP_05 #302 RT: 3.06 AV: 1 NL: 2.52E9
T: FTMS + p ESI Full ms [105.0000-1000.0000]
259.08240
z=1
157.03513
z=?
245.08354
z=?
281.06418
z=1
367.10419
z=?
517.15778
z=1
570.06921
z=1
680.42621
z=?
791.00531
z=?
827.38910
z=?
891.85718
z=?
m/z

2.4 Crystallography of compound **18**

Figure S1 below is a copy of Figure 2 from the main document and is repeated here for context.

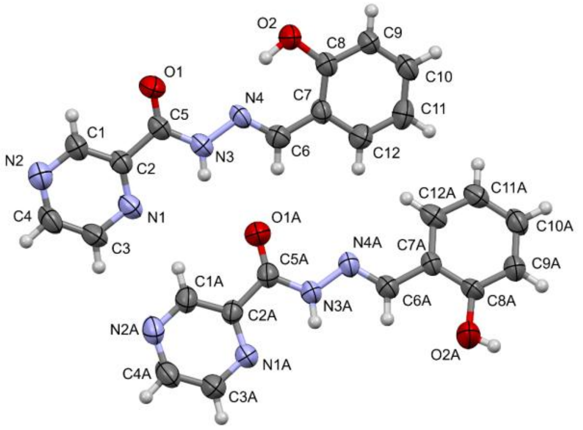

Figure S1 (identical with Figure 2 in the main document)**.** Thermal ellipsoid diagram of **18**, with the arbitrary atom-numbering scheme. Displacement ellipsoids are drawn at the 50% probability level. In the following text, the upper molecule is designated as I, and the lower molecule as A.

The best mean planes of the two molecules in the ASU form an angle of 20.6°. The difference in orientation of the phenol moiety reflects a difference in the interactions established by the hydroxyl group. In molecule I, the OH (O2-H2) is engaged in an intramolecular H-bond with N4 of the hydrazone. Conversely, in molecule A, the OH (O2A-H2A) forms a strong intermolecular H-bond with the carbonyl oxygen (O1) of a neighbouring molecule I, belonging to an adjacent ASU. Interestingly, the proximity of the hydroxyl group (O2-H2) of the same interacting molecule exerts a pulling force on the OH hydrogen (H2A), which appears to be slightly disordered. The O2A-H2A···O1 H-bond leads to the formation of linear assemblies of molecular dimers, extending along the *b* axis. The packing is further consolidated by weaker CπH···X interactions (with X = N, O), mainly connecting the aromatic rings along the *a* and *c* axes. The presence of the pyrazine and phenyl ring also determines the formation of parallel-displaced π-π stacking interactions. In detail, phenyl-pyrazine contacts (centroid-centroid distance: 3.95 Å) and phenyl-phenyl interactions (centroid-centroid distance: 4.34 Å) connect the molecules along the *a* and *c* axes, respectively. The packing and the main interactions are visualized in Figure S2; the complete account of the H-bonds is available in Table S4.

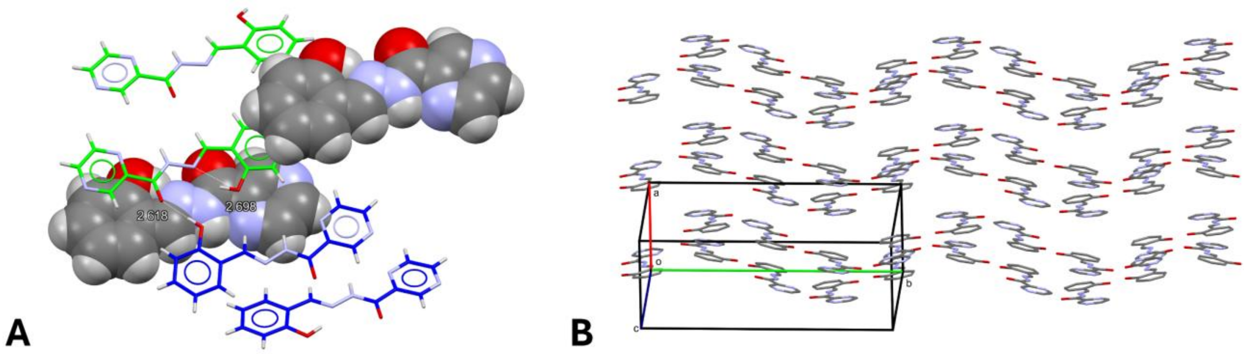

Figure S2. A - Stick and spacefill model of **18** showing the main H-bonds and stacking interactions in an arbitrary view. The green and blue pairs of molecules make up different ASUs. **B -** Crystal packing viewed along the *c* axis, showing the dimers extending in chains along the *b* axis. Hydrogens are omitted for clarity.

Table S4. H-bond geometry (D: Donor, A: Acceptor) in the crystal of **18**

Bond	D-H/Å	H···A/Å	D···A/Å	D-H···A/°
O2-H2···N4^{I}	0.92(4)	1.85(4)	2.698(4)	151(3)
C1A-H1A···N1^{I}	0.930(4)	2.788(3)	3.498(5)	134.0(3)
C3-H3···N2A^{I}	0.930(4)	2.675(4)	3.487(6)	146.3(3)
O2A-H2A···O1^{II}	0.820(3)	1.801(3)	2.617(4)	173.9(3)
C9-H9···N1A^{III}	0.930(4)	2.970(3)	3.875(5)	164.9(3)
C10-H10···N2^{IV}	0.930(4)	2.716(3)	3.607(5)	160.6(3)
C10A-H10A···N2A^{IV}	0.930(4)	2.693(3)	3.596(5)	164.1(3)
C11A-H11A···O2^{V}	0.930(4)	2.658(3)	3.472(5)	146.6(3)
C1-H1···O1A^{VI}	0.930(4)	2.772(3)	3.467(5)	132.3(3)

Equivalent positions: ^{I}x, y, z; ^{II} 1-x, y-1/2, 3/2-z; ^{III} 1-x, y+1/2, 3/2-z; ^{IV} x-1, y, z-1; ^{V} 1-x, -y, 1-z; ^{VI} 2-x, -y, 2-z.

The analysis of the Hirshfeld surface (HS) substantially confirmed the previous observations. The HS was computed for both molecules in the ASU, showing minimal differences. For ease of discussion, the following brief description refers to the HS of molecule I (V = 273.28 Å^3, A = 273.93 Å^2, G = 0.743, Ω = 0.418). The d_{norm} property (Figure S3A) was visualized with a red-blue-white color scheme based on the length of the intermolecular contacts with respect to the sum of the van der Waals radii. The presence of an intense red spot indicated the presence of a short-range contact, corresponding to the H-bond that leads to the formation of the dimers. The presence of the short-range O···H contact was further visualized by the long spike protruding towards the lower left corner of the two-dimensional (2D) fingerprint plot of the HS (Figure S3B). The central area of the graph also confirmed the contribution of stacking interactions to the packing, further substantiated by the large flat region evidenced by the curvedness plot (Figure S3C). Finally, the calculation of the enrichment ratios confirmed the prominence of O···H (1.83), C···C (1.37), and C···N (1.32) interactions in the contact network. Non-specific H···H contacts were also found to be enriched (1.11).

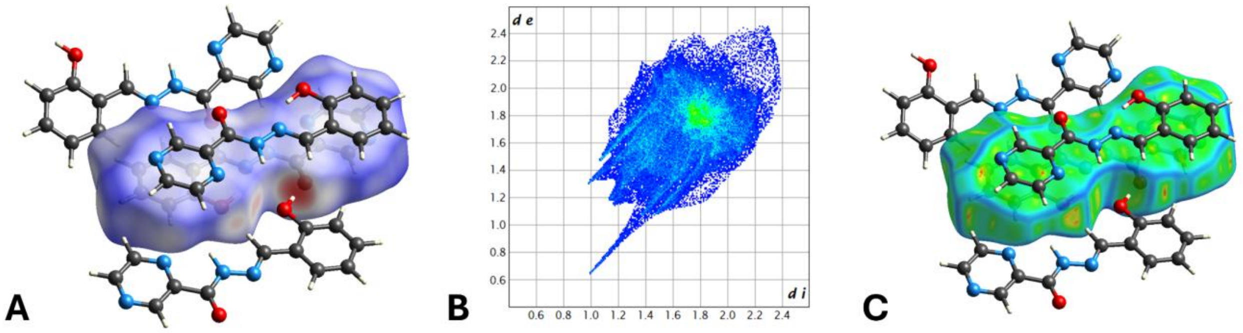

Figure S3. A - Hirshfeld surface of **18** (molecule I) mapped over d_{norm} with a fixed color scale in the range -0.7536 au (red) – 1.2295 au (blue), based on the length of the intermolecular contacts with respect to the sum of the van der Waals radii (red: shorter; blue: longer; white: same). **B -** 2D fingerprint plot of the HS of **18** (molecule I). The graphical representation provides a summary of the frequency of each combination of d_e (distances from the HS to the nearest nucleus outside the surface) and d_i (distances from the HS to the nearest nucleus inside the surface) across the HS. Points with a lower contribution to the surface are colored blue, while green indicates larger contributions. **C -** Curvedness plot of **18** (molecule I); green represents flat regions, while blue indicates edges (color scale: -3.5688 au – 0.4114 au).

2.5 Results of incubation of compound **18** with human plasma

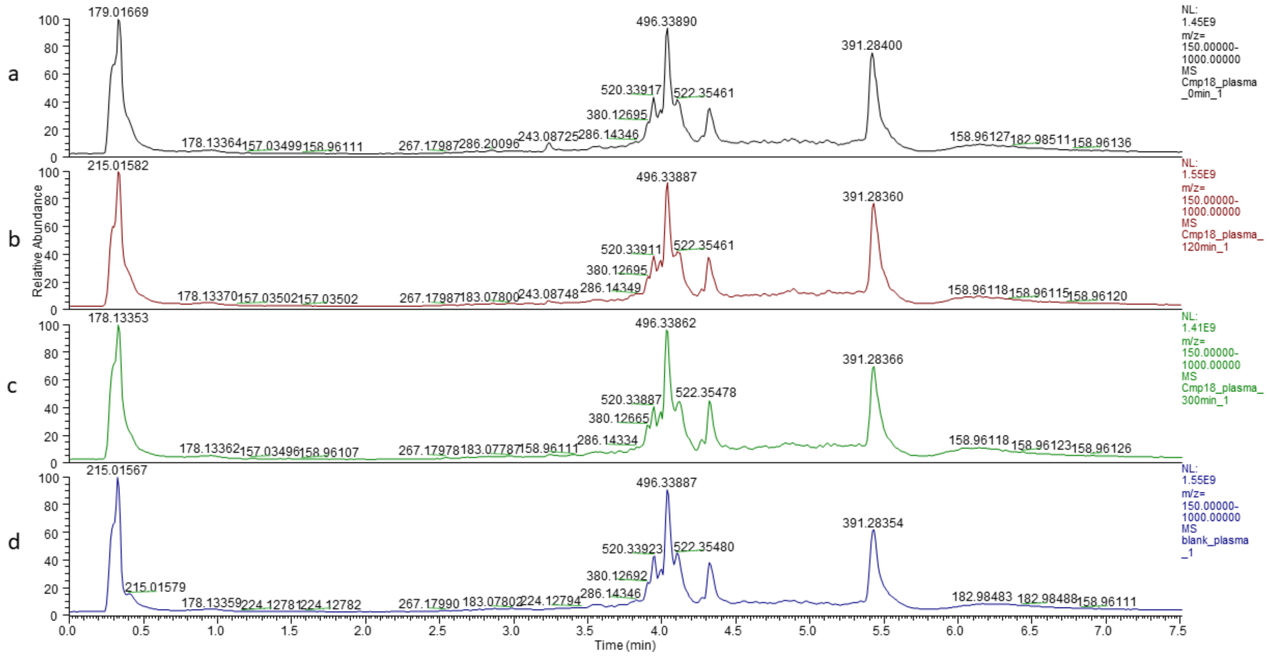

Figure S4. MS spectra of compound **18** after incubation with human plasma. a) incubation after 0 min, b) after 120 min, and c) after 300 min. d) MS spectrum of blank plasma without compound **18**.

2.6 Results of *in vitro* antimicrobial screening

2.6.1 Antimycobacterial activity

Table S5. Full results of antimycobacterial activity expressed as MIC in µg/mL

		MIC (µg/mL)			
**Compound**	**logP**	***M. tuberculosis* H37Ra**	***M. tuberculosis* H37Rv***	***M. smegmatis***	***M. aurum***
**PCH**	1.56	>500		>500	>500
**1**	1.67	N/A		N/A	N/A
**2**	1.67	>250		>250	>250
**3**	1.67	>250		>250	>250
**4**	1.94	>500		>500	>500
**5**	1.94	>500		>500	>500
**6**	1.94	>500		>500	>500
**7**	1.27	>500		>500	>500
**8**	1.27	>125		>125	>125
**9**	1.27	>500		>500	>500
**10**	2.79	>500		>500	>500
**11**	0.52	N/A		N/A	N/A
**12**	0.52	>500		>500	>500
**13**	0.52	>125		>125	>125
**14**	0.99	>500		>500	>500
**15**	0.60	>500		>500	>500
**16**	0.99	>125		>125	>125
**17**	0.99	>250		>250	>250
**18**	0.72	62.5	25	>500	31.25
**19**	0.72	>500		>500	>500
**20**	0.72	>500		>500	>500
**21**	0.33	15.625	25	>500	15.625
**22**	0.33	>500		>500	>500
**23**	0.13	>500		>500	>500
**24**	0.22	250		>500	125
**25**	0.22	N/A		N/A	N/A
**26**	0.22	>250		>250	>250
**27**	0.27	>250		>250	>250
**28**	0.50	250		>500	125
**29**	1.10	>500		>500	>500
**30**	1.43	>500		>500	>500
**31**	2.58	>250		>250	>250
**32**	1.00	>250		>250	>250
**33**	0.52	>500		>500	>500
**INH**	-	0.25		31.25	1.98
**RIF**	-	0.003125		25	0.39
**CIP**	-	0.25		0.125	0.015625

* Activity tested only for compounds that showed activity in the initial screening on *M. tuberculosis* H37Ra. INH – isoniazid, RIF – rifampicin. CIP- ciprofloxacin. N/A – Not Available.

2.6.2 Antibacterial activity

Table S6. Full results of antibacterial activity expressed as MIC in µM

	**MIC [µM]**															
	**SA**		**MRSA**		**SE**		**EF**		**EC**		**KP**		**ACI**		**PA**	
**No.**	24h	48h	24h	48h	24h	48h	24h	48h	24h	48h	24h	48h	24h	48h	24h	48h
**PCH**	>500	>500	>500	>500	>500	>500	>500	>500	>500	>500	>500	>500	>500	>500	>500	>500
**1**	>500	>500	>500	>500	>500	>500	>500	>500	>500	>500	>500	>500	>500	>500	>500	>500
**2**	N/A	N/A	N/A	N/A	N/A	N/A	N/A	N/A	N/A	N/A	N/A	N/A	N/A	N/A	N/A	N/A
**3**	>500	>500	>500	>500	>500	>500	>500	>500	>500	>500	>500	>500	>500	>500	>500	>500
**4**	>500	>500	>500	>500	>500	>500	>500	>500	>500	>500	>500	>500	>500	>500	>500	>500
**5**	>500	>500	>500	>500	>500	>500	>500	>500	>500	>500	>500	>500	>500	>500	>500	>500
**6**	>125	>125	>125	>125	>125	>125	>125	>125	>125	>125	>125	>125	>125	>125	>125	>125
**7**	>500	>500	>500	>500	>500	>500	>500	>500	>500	>500	>500	>500	>500	>500	>500	>500
**8**	>125	>125	>125	>125	>125	>125	>125	>125	>125	>125	>125	>125	>125	>125	>125	>125
**9**	>500	>500	>500	>500	>500	>500	>500	>500	>500	>500	>500	>500	>500	>500	>500	>500
**10**	>500	>500	>500	>500	>500	>500	>500	>500	>500	>500	>500	>500	>500	>500	>500	>500
**11**	>250	>250	>250	>250	>250	>250	>250	>250	>250	>250	>250	>250	>250	>250	>250	>250
**12**	>125	>125	>125	>125	>125	>125	>125	>125	>125	>125	>125	>125	>125	>125	>125	>125
**13**	>250	>250	**125**	**250**	>250	>250	>250	>250	>250	>250	>250	>250	>250	>250	>250	>250
**14**	N/A	N/A	N/A	N/A	N/A	N/A	N/A	N/A	N/A	N/A	N/A	N/A	N/A	N/A	N/A	N/A
**15**	>500	>500	>500	>500	>500	>500	>500	>500	>500	>500	>500	>500	>500	>500	>500	>500
**16**	>250	>250	>250	>250	>250	>250	>250	>250	>250	>250	>250	>250	>250	>250	>250	>250
**17**	>500	>500	>500	>500	>500	>500	>500	>500	>500	>500	>500	>500	>500	>500	>500	>500
**18**	>500	>500	>500	>500	>500	>500	>500	>500	>500	>500	>500	>500	>500	>500	>500	>500
**19**	>500	>500	>500	>500	>500	>500	>500	>500	>500	>500	>500	>500	>500	>500	>500	>500
**20**	>500	>500	>500	>500	>500	>500	>500	>500	>500	>500	>500	>500	>500	>500	>500	>500
**21**	>500	>500	>500	>500	>500	>500	>500	>500	>500	>500	>500	>500	>500	>500	>500	>500
**22**	>500	>500	>500	>500	>500	>500	>500	>500	>500	>500	>500	>500	>500	>500	>500	>500
**23**	>500	>500	>500	>500	>500	>500	>500	>500	>500	>500	>500	>500	>500	>500	>500	>500
**24**	>250	>250	>250	>250	>250	>250	>250	>250	>250	>250	>250	>250	>250	>250	>250	>250
**25**	>125	>125	>125	>125	>125	>125	>125	>125	>125	>125	>125	>125	>125	>125	>125	>125
**26**	>500	>500	>500	>500	>500	>500	>500	>500	>500	>500	>500	>500	>500	>500	>500	>500
**27**	>500	>500	>500	>500	>500	>500	>500	>500	>500	>500	>500	>500	>500	>500	>500	>500
**28**	>500	>500	>500	>500	>500	>500	>500	>500	>500	>500	>500	>500	>500	>500	>500	>500
**29**	>500	>500	>500	>500	>500	>500	>500	>500	>500	>500	>500	>500	>500	>500	>500	>500
**30**	>500	>500	>500	>500	>500	>500	>500	>500	>500	>500	>500	>500	>500	>500	>500	>500
**31**	>500	>500	>500	>500	>500	>500	>500	>500	>500	>500	>500	>500	>500	>500	>500	>500
**32**	>500	>500	>500	>500	>500	>500	>500	>500	>500	>500	>500	>500	>500	>500	>500	>500
**33**	>500	>500	>500	>500	>500	>500	>500	>500	>500	>500	>500	>500	>500	>500	>500	>500

SA - *Staphylococcus aureus*, MRSA - methicillin-resistant *Staphylococcus aureus*, SE - *Staphylococcus epidermidis*, EF - *Enterococcus faecalis*, EC - *Escherichia coli*, KP - *Klebsiella pneumoniae*, ACI - *Acinetobacter baumannii*, PA - *Pseudomonas aeruginosa*

2.6.3 Antifungal activity

Table S7. Full results of antifungal activity expressed as MIC in µM

	MIC (µM)															
	CA		CK		CP		CT		AF		AFla		LC		TI	
**No.**	24h	48h	24h	48h	24h	48h	24h	48h	24h	48h	24h	48h	24h	48h	72h	120h
**PCH**	>500	>500	>500	>500	>500	>500	>500	>500	>500	>500	>500	>500	>500	>500	>500	>500
**1**	>500	>500	>500	>500	>500	>500	>500	>500	>500	>500	>500	>500	>500	>500	>500	>500
**2**	N/A	N/A	N/A	N/A	N/A	N/A	N/A	N/A	N/A	N/A	N/A	N/A	N/A	N/A	N/A	N/A
**3**	>125	>125	>125	>125	>125	>125	>125	>125	>125	>125	>125	>125	>125	>125	>125	>125
**4**	>500	>500	>500	>500	>500	>500	>500	>500	>500	>500	>500	>500	>500	>500	>500	>500
**5**	>500	>500	>500	>500	>500	>500	>500	>500	>500	>500	>500	>500	>500	>500	>500	>500
**6**	>125	>125	>125	>125	>125	>125	>125	>125	>125	>125	>125	>125	>125	>125	>125	>125
**7**	>500	>500	>500	>500	>500	>500	>500	>500	>500	>500	>500	>500	>500	>500	>500	>500
**8**	>125	>125	>125	>125	>125	>125	>125	>125	>125	>125	>125	>125	>125	>125	>125	>125
**9**	>500	>500	>500	>500	>500	>500	>500	>500	>500	>500	>500	>500	>500	>500	>500	>500
**10**	>125	>125	>125	>125	>125	>125	>125	>125	>125	>125	>125	>125	>125	>125	>125	>125
**11**	>125	>125	>125	>125	>125	>125	>125	>125	>125	>125	>125	>125	>125	>125	>125	>125
**12**	>125	>125	>125	>125	>125	>125	>125	>125	>125	>125	>125	>125	>125	>125	>125	>125
**13**	>125	>125	>125	>125	>125	>125	>125	>125	>125	>125	>125	>125	>125	>125	>125	>125
**14**	N/A	N/A	N/A	N/A	N/A	N/A	N/A	N/A	N/A	N/A	N/A	N/A	N/A	N/A	N/A	N/A
**15**	>500	>500	>500	>500	>500	>500	>500	>500	>500	>500	>500	>500	>500	>500	>500	>500
**16**	>250	>250	>250	>250	>250	>250	>250	>250	>250	>250	>250	>250	>250	>250	>250	>250
**17**	>500	>500	>500	>500	>500	>500	>500	>500	>500	>500	>500	>500	>500	>500	>500	>500
**18**	**250**	500	**125**	**250**	>500	>500	**500**	>500	>500	>500	>500	>500	**250**	**500**	**62.5**	**250**
**19**	>500	>500	>500	>500	>500	>500	>500	>500	>500	>500	>500	>500	>500	>500	>500	>500
**20**	>500	>500	>500	>500	>500	>500	>500	>500	>500	>500	>500	>500	>500	>500	>500	>500
**21**	**250**	>250	**62.5**	**125**	250	>250	**125**	>250	>250	>250	>250	>250	**250**	>250	**62.5**	**125**
**22**	>125	>125	>125	>125	>125	>125	>125	>125	>125	>125	>125	>125	>125	>125	>125	>125
**23**	>500	>500	>500	>500	>500	>500	>500	>500	>500	>500	>500	>500	>500	>500	>500	>500
**24**	>250	>250	>250	>250	>250	>250	>250	>250	>250	>250	>250	>250	>250	>250	>250	>250
**25**	>125	>125	>125	>125	>125	>125	>125	>125	>125	>125	>125	>125	>125	>125	>125	>125
**26**	>500	>500	>500	>500	>500	>500	>500	>500	>500	>500	>500	>500	>500	>500	>500	>500
**27**	>500	>500	>500	>500	>500	>500	>500	>500	>500	>500	>500	>500	>500	>500	>500	>500
**28**	**500**	**500**	**500**	**500**	>500	>500	>500	>500	**500**	**500**	**500**	**500**	**250**	**250**	**250**	**250**
**29**	>500	>500	**500**	**500**	>500	>500	>500	>500	>500	>500	>500	>500	>500	>500	>500	>500
**30**	>500	>500	>500	>500	>500	>500	>500	>500	>500	>500	>500	>500	>500	>500	>500	>500
**31**	>500	>500	>500	>500	>500	>500	>500	>500	>500	>500	>500	>500	>500	>500	>500	>500
**32**	>500	>500	>500	>500	>500	>500	>500	>500	>500	>500	>500	>500	>500	>500	>500	>500
**33**	>500	>500	>500	>500	>500	>500	>500	>500	>500	>500	>500	>500	>500	>500	>500	>500

CA - *Candida albicans*, CK - *Candida krusei*, CP - *Candida parapsilosis*, CT - *Candida tropicalis*, AF - *Aspergillus fumigatus*, AFla - *Aspergillus flavus*, LC - *Lichtheimia corymbifera*, TI - *Trichophyton interdigitale*

2.7 Mechanism of action studies

2.7.1 Results of enoyl-ACP reductase (InhA) inhibition assay

Table S8. Results of mycobacterial enoyl-ACP reductase inhibition as residual activity at 100 µM

Compound	Residual activity @ 100 µM
**14**	106 %
**15**	100%
**16**	98%
**17**	96%
**18**	73%
**19**	77%
**20**	100%
**21**	120%
**22**	100%

control compound (J. Med. Chem. 2015, 58, 613-624) (IC_{50} = 0.068 µM)

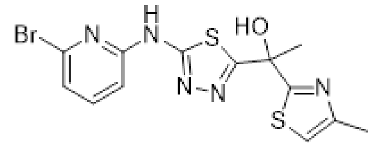

Chemical Formula: $C_{13}H_{12}BrN_5OS_2$
Exact Mass: 396,9667
Molecular Weight: 398,2970

2.7.2 Results of isocitrate lyase (ICL) inhibition assay

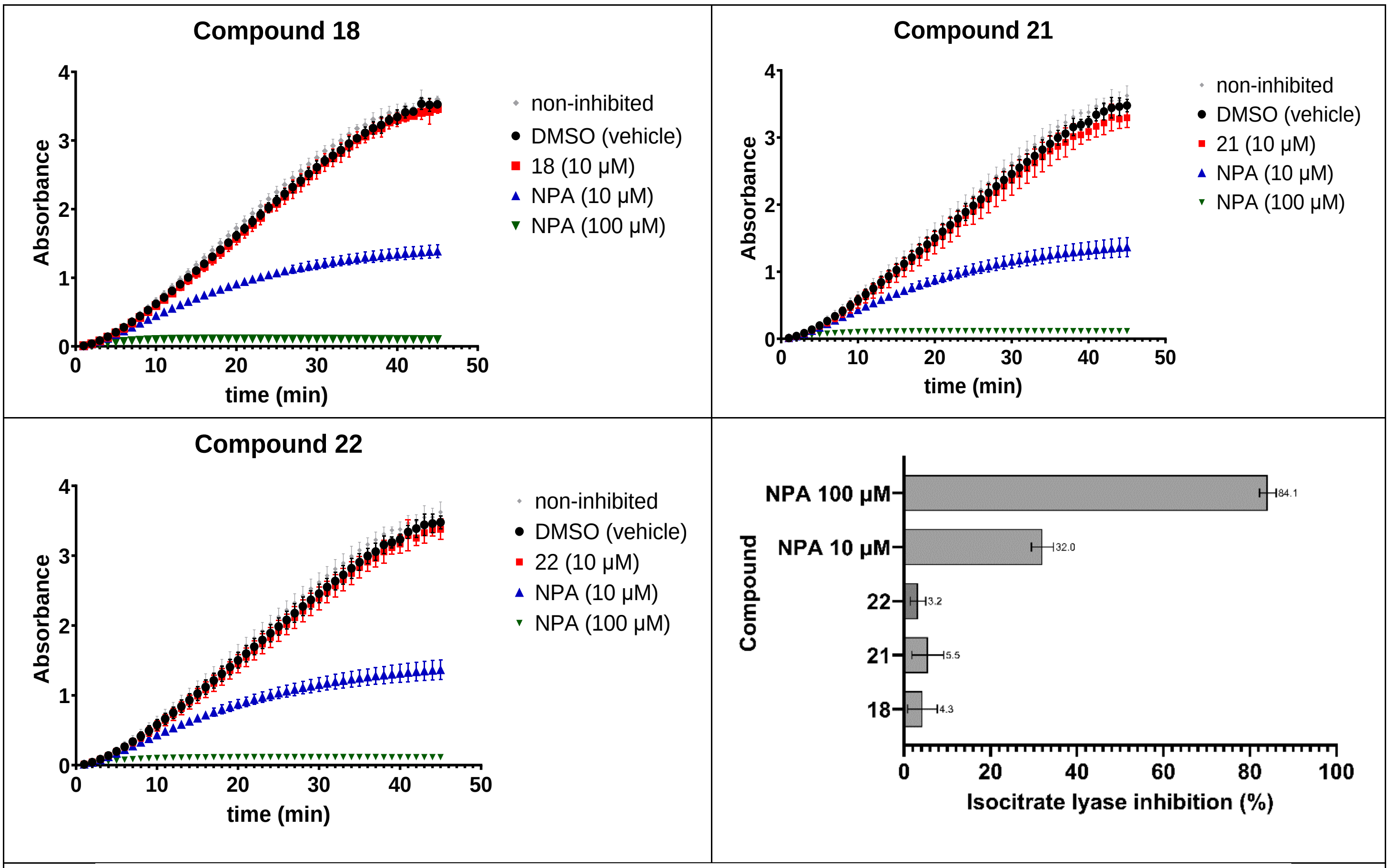

18			21			22			NPA 10 μM			NPA 100 μM		
Mean	SD	N	Mean	SD	N	Mean	SD	N	Mean	SD	N	Mean	SD	N
4.252	3.442	3	5.494	3.676	3	3.221	1.797	3	32.039	2.563	6	84.148	1.931	6

Figure S5. Inhibition of mycobacterial isocitrate lyase (ICL) by compounds **18**, **21**, and **22** at 10 μM compared with the standard inhibitor 3-nitropropanoic acid (NPA). Non-inhibited means untreated control without addition of the vehiculum (DMSO).

3. References

[1] S. G. Franzblau, R. S. Witzig, J. C. McLaughlin, P. Torres, G. Madico, A. Hernandez, M. T. Degnan, M. B. Cook, V. K. Quenzer, R. M. Ferguson, R. H. Gilman, "Rapid, low-technology MIC determination with clinical Mycobacterium tuberculosis isolates by using the microplate Alamar Blue assay", *J Clin Microbiol* **1998**, *36*, 362-366. doi: 10.1128/JCM.36.2.362-366.1998

[2] European Committee for Antimicrobial Susceptibility Testing (EUCAST) of the European Society of Clinical Microbiology and Infectious Diseases (ESCMID). "Determination of minimum inhibitory concentrations (MICs) of antibacterial agents by broth dilution.", *Clin Microbiol Infect* **2003**, *9*, 1-7. doi: n/a

[3] Arendrup, M.C.; Meletiadis, J.; Mouton, J.W.; Lagrou, K.; Hamal, P.; Guinea, J.; Subcommittee on Antifungal Susceptibility Testing (AFST) of the ESCMID European Committee for Antimicrobial Susceptibility Testing (EUCAST). Method for the Determination of Broth Dilution Minimum Inhibitory Concentrations of Antifungal agents for Yeasts. EUCAST Definitive Document E.Def 7.3.1. 2017. Available online: http://www.eucast.org/asto_ungi/methodsinantifungalsusceptibilitytesting/susceptibility_testing_of_yeasts/ (accessed on 10 June 2025).

[4] Arendrup, M.C.; Meletiadis, J.; Mouton, J.W.; Lagrou, K.; Hamal, P.; Guinea, J.; Subcommittee on Antifungal Susceptibility Testing (AFST) of the ESCMID European Committee for Antimicrobial Susceptibility Testing (EUCAST). Method for the Determination of Broth Dilution Minimum Inhibitory Concentrations of Antifungal agents for Conidia Forming Moulds. EUCAST Definitive Document E.Def 9.3.1. 2017. Available online: http://www.eucast.org/asto_ungi/methodsinantifungalsusceptibilitytesting/susceptibility_testing_of_moulds/ (accessed on 10 June 2025).

[5] SMART & SAINT Software Reference Manual, Version 6.45. Bruker Analytical X-Ray Systems, Inc., Madison, 2003.

[6] Sheldrick, G.M. (2010) SADABS, Program for Empirical Absorption Correction of Area Detector Data. University of Gottingen, Gottingen, Germany.

[7] M. C. Burla, R. Caliandro, B. Carrozzini, G. L. Cascarano, C. Cuocci, C. Giacovazzo, M. Mallamo, A. Mazzone, G. Polidori, "Crystal structure determination and refinement", *J Appl Crystallogr* **2015**, *48*, 306-309. doi: 10.1107/S1600576715001132

[8] G. M. Sheldrick, "Crystal structure refinement with SHELXL", *Acta Crystallogr C Struct Chem* **2015**, *71*, 3-8. doi: 10.1107/S2053229614024218

[9] R. Agoro, C. Mura, "Iron Supplementation Therapy, A Friend and Foe of Mycobacterial Infections?", *Pharmaceuticals (Basel)* **2019**, *12*, 75. doi: 10.3390/ph12020075

[10] M. Nardelli, "PARST95 – an update to PARST: a system of Fortran routines for calculating molecular structure parameters from the results of crystal structure analyses", *J Appl Crystallogr* **1995**, *28*, 659-659. doi: 10.1107/s0021889895007138

[11] C. F. Macrae, I. Sovago, S. J. Cottrell, P. T. A. Galek, P. McCabe, E. Pidcock, M. Platings, G. P. Shields, J. S. Stevens, M. Towler, P. A. Wood, "Mercury 4.0: from visualization to analysis, design and prediction", *J Appl Crystallogr* **2020**, *53*, 226-235. doi: 10.1107/S1600576719014092

[12] P. R. Spackman, M. J. Turner, J. J. McKinnon, S. K. Wolff, D. J. Grimwood, D. Jayatilaka, M. A. Spackman, "CrystalExplorer: a program for Hirshfeld surface analysis, visualization and quantitative analysis of molecular crystals", *J Appl Crystallogr* **2021**, *54*, 1006-1011. doi: 10.1107/S1600576721002910

[13] B. Guillot, E. Enrique, L. Huder, C. Jelsch, "MoProViewer: a tool to study proteins from a charge density science perspective", *Acta Crystallographica a-Foundation and Advances* **2014**, *70*, C279-C279. doi: 10.1107/S2053273314097204

[14] H. Jansova, J. Kubes, P. Reimerova, P. Sterbova-Kovarikova, J. Roh, T. Simunek, "2,6-Dihydroxybenzaldehyde Analogues of the Iron Chelator Salicylaldehyde Isonicotinoyl Hydrazone: Increased Hydrolytic Stability and Cytoprotective Activity against Oxidative Stress", *Chem Res Toxicol* **2018**, *31*, 1151-1163. doi: 10.1021/acs.chemrestox.8b00165

[15] S. Parikh, D. P. Moynihan, G. Xiao, P. J. Tonge, "Roles of tyrosine 158 and lysine 165 in the catalytic mechanism of InhA, the enoyl-ACP reductase from Mycobacterium tuberculosis", *Biochemistry* **1999**, *38*, 13623-13634. doi: 10.1021/bi990529c
[16] M. Kratky, J. Vinsova, E. Novotna, J. Mandikova, F. Trejtnar, J. Stolakova, "Antibacterial activity of salicylanilide 4-(trifluoromethyl)-benzoates", *Molecules* **2013**, *18*, 3674-3688. doi: 10.3390/molecules18043674
[17] M. M. Bradford, "A rapid and sensitive method for the quantitation of microgram quantities of protein utilizing the principle of protein-dye binding", *Anal Biochem* **1976**, *72*, 248-254. doi: 10.1016/0003-2697(76)90527-3
[18] G. H. Dixon, H. L. Kornberg, "Assay Methods for Key Enzymes of the Glyoxylate Cycle", *Biochemical Journal* **1959**, *72*, P3-P3. doi: n/a
[19] V. Finger, T. Kucera, R. Kafkova, L. Muckova, R. Dolezal, J. Kubes, M. Novak, L. Prchal, L. Lakatos, M. Andrs, M. Hympanova, J. Marek, M. Kufa, V. Spiwok, O. Soukup, E. Mezeiova, J. Janousek, L. Nevosadova, M. Benkova, R. R. A. Kitson, M. Kratky, S. Bosze, K. Mikusova, R. Hartkoorn, J. Roh, J. Korabecny, "2,6-Disubstituted 7-(naphthalen-2-ylmethyl)-7H-purines as a new class of potent antitubercular agents inhibiting DprE1", *Eur J Med Chem* **2023**, *258*, 115611. doi: 10.1016/j.ejmech.2023.115611
[20] M. Novak, B. Svobodova, J. Konecny, A. Kuratkova, L. Nevosadova, L. Prchal, J. Korabecny, V. M. Lauschke, O. Soukup, R. Kucera, "UHPLC-Orbitrap study of the first phase tacrine in vitro metabolites and related Alzheimer's drug candidates using human liver microsomes", *J Pharm Biomed Anal* **2023**, *224*, 115154. doi: 10.1016/j.jpba.2022.115154
[21] X. F. Shi, C. C. Yuan, "'-(2-chlorobenzylidene)pyrazine-2-carbohydrazide", *Acta Crystallographica Section E-Structure Reports Online* **2006**, *62*, O5232-O5233. doi: 10.1107/S1600536806043212
[22] T. C. Baddeley, R. A. Howie, C. H. D. Lima, C. R. Kaiser, M. V. N. de Souza, J. L. Wardell, S. M. S. V. Wardell, "(Pyrazinecarbonyl)hydrazone halobenzaldehydes: supramolecular arrays generated by face to face stacking of ribbons, formed from C-H-O interactions", *Zeitschrift Fur Kristallographie* **2009**, *224*, 506-514. doi: 10.1524/zkri.2009.1182
[23] F. M. Vergara, C. H. Lima, M. Henriques, A. L. Candea, M. C. Lourenco, L. Ferreira Mde, C. R. Kaiser, M. V. de Souza, "Synthesis and antimycobacterial activity of N'-[(E)-(monosubstituted-benzylidene)]-2-pyrazinecarbohydrazide derivatives", *Eur J Med Chem* **2009**, *44*, 4954-4959. doi: 10.1016/j.ejmech.2009.08.009
[24] C. C. Yuan, L. Wu, Z. Y. Xing, X. F. Shi, "4-chlorobenzaldehyde (pyrazin-2-ylcarbonyl)hydrazone", *Acta Crystallographica Section E-Structure Reports Online* **2006**, *62*, O3274-O3275. doi: 10.1107/S1600536806026006
[25] M. Ahmad, S. Hameed, M. N. Tahir, M. Anwar, M. Israr, "N'-[(E)-3-Bromo-benzyl-idene]pyrazine-2-carbohydrazide", *Acta Crystallogr Sect E Struct Rep Online* **2013**, *69*, o1635. doi: 10.1107/S1600536813027426
[26] Y. K. da Silva, C. V. Augusto, M. L. de Castro Barbosa, G. M. de Albuquerque Melo, A. C. de Queiroz, T. de Lima Matos Freire Dias, W. B. Junior, E. J. Barreiro, L. M. Lima, M. S. Alexandre-Moreira, "Synthesis and pharmacological evaluation of pyrazine N-acylhydrazone derivatives designed as novel analgesic and anti-inflammatory drug candidates", *Bioorg Med Chem* **2010**, *18*, 5007-5015. doi: 10.1016/j.bmc.2010.06.002
[27] R. A. Howie, C. H. D. Lima, C. R. Kaiser, M. V. N. de Souza, J. L. Wardell, S. M. S. V. Wardell, "Structures of (pyrazinecarbonyl)hydrazones of substituted benzaldehydes: supramolecular arrangements generated by various intermolecular contacts", *Zeitschrift Fur Kristallographie-Crystalline Materials* **2010**, *225*, 19-28. doi: 10.1524/zkri.2010.1209
[28] M. V. de Souza, C. H. da Silva Lima, J. L. Wardell, S. M. Wardell, E. R. Tiekink, "N'-[(E)-2-Meth-oxy-benzyl-idene]pyrazine-2-carbohydrazide", *Acta Crystallogr Sect E Struct Rep Online* **2011**, *67*, o1714-1715. doi: 10.1107/S1600536811022938
[29] M. Abdel-Aziz, H. M. Abdel-Rahman, "Synthesis and anti-mycobacterial evaluation of some pyrazine-2-carboxylic acid hydrazide derivatives", *Eur J Med Chem* **2010**, *45*, 3384-3388. doi: 10.1016/j.ejmech.2010.04.025

[30] I. d. S. B. Felipe A. R. Rodrigues, Bruno C. Cavalcanti, Claudia Ó Pessoa, Alessandra C. Pinheiro, Camilo H. S. Lima, Marcus V. N. de Souza, "BIOLOGICAL EVALUATION OF PYRAZINAMIDE DERIVATIVES AS AN ANTICANCER CLASS", *European Chemical Bulletin* **2014**, *3*, 358-361. doi: 10.17628/ECB.2014.3.358
[31] M. D. Ferreira, A. L. P. Candea, M. D. M. D. Henriques, C. R. Kaiser, C. H. D. Lima, M. V. N. de Souza, "Synthesis and Cytotoxic Evaluation of Disubstituted N-Acylhydrazones Pyrazinecarbohydrazide Derivatives", *Letters in Drug Design & Discovery* **2010**, *7*, 275-280. doi: n/a
[32] S. Bond, P. J. Huggins, J. G. Parsons. "Preparation of hydrazides for treating diseases modulated by reducing zinc and/or iron". WO2022040747, 2022.
[33] D. S. Goldfarb. "Method using lifespan-altering compounds for altering the lifespan of eukaryotic organisms, and screening for such compounds". US20090163545, 2009.
